# Long-term impact of invasive meningococcal disease in children: SEINE study protocol

**DOI:** 10.1371/journal.pone.0268536

**Published:** 2022-05-26

**Authors:** Alexiane Baloche, Camille Jung, Michael Levy, Annie Elbez-Rubinstein, Stéphane Béchet, Ines Layouni, Geneviève Monguillot, Muhamed Kheir Taha, Robert Cohen, Corinne Levy

**Affiliations:** 1 PhD student, Grenoble-Alpes University, Grenoble, France; 2 UR 4129 P2S Parcours Santé Systémique, Lyon, France; 3 Centre Hospitalier Intercommunal, Clinical Research Center, Créteil, France; 4 Université Paris Est, IMRB-GRC GEMINI, Créteil, France; 5 Paediatric Intensive Care Unit, Robert-Debré University Hospital, Assistance Publique-Hôpitaux de Paris, Paris, France; 6 Institut Pasteur, National Reference Center for Meningococcus, Paris, France; 7 ACTIV (Association Clinique et Thérapeutique Infantile du Val de Marne), Créteil, France; 8 French Pediatric Infectious Disease Group, GPIP, Créteil, France; Public Health England, UNITED KINGDOM

## Abstract

**Introduction:**

Invasive meningococcal disease (IMD) is still an important cause of mortality in children and survivors can have significant long-term disabling sequelae. There are few prospective studies looking at the long term neuropsychological and developmental consequences of IMD in surviving children, and the rate of sequelae may be underestimated. The SEINE study aims to have a more reliable estimate of the real rate of sequelae by assessing the long-term physical, neuropsychological, learning disorders and sensory sequelae of IMD in children and adolescents and by assessing the post-traumatic stress in parents.

**Methods and analysis:**

The SEINE study is a multicentre, prospective, non-randomized, interventional study based on the French bacterial meningitis surveillance network. The study will include 100 children aged from birth to 15 years old, hospitalized in a Paris area paediatric ward for a meningococcal meningitis or a purpura fulminans between 2010 and 2019. The first outcome will assess long-term sequelae (physical, neurological, or sensory) measured by a general clinical and neurological examination, a neurocognitive assessment, learning development, a pure tone audiometry and an ophthalmic examination. The second outcome will assess the long-term post-traumatic stress in parents measured by the Impact of Event Scare Revised questionnaire.

**Perspectives:**

By providing a better estimation of the rate of sequelae in children and offering an adapted follow-up of these children, we believe that the SEINE study will help to improve the management of patients surviving IMD.

**Trial registration number:**

NCT04685850.

## Introduction

Invasive meningococcal disease (IMD) is an important health concern in many countries. In Europe, in 2017, the overall incidence was 0.6 cases/ 100 000 (ranged between 0.1 and 2.5/100 000) [1]. In France, the overall incidence of IMD in 2019 was 0.76 cases /100 000 inhabitants but is significantly higher among children < 1 year old (9.1/ 100 000 in 2019) and young adults aged between 15 and 24 years old (1.2/100 000 in 2019) [2]. Over the last ten years, the implementation of various meningococcal vaccines (MenC, Men ACWY and MenB) in many countries had a major impact on the epidemiology of IMD with a decrease in incidence and mortality [3]. Changing epidemiology of IMD has led to variable strategies of vaccination with variable vaccine uptake in different countries [4]. Despite advances in medical and critical care, IMD continues to have a case fatality rate of 9% for meningococcal meningitis (MM) and up to 23% for purpura fulminans (PF) [2, 5]. In addition, survivors of MM or PF are at risk of long-term disabling sequelae and impaired quality of life [6]. To date, the short-term sequelae after hospitalization for IMD are well known, varying between 10 to 40% [7–10]. Sequelae are most often cutaneous (skin necrosis with more or less extensive skin loss, which may require a skin graft), orthopaedic (amputations), neurological (focal neurological deficits, epilepsy, spasticity, deafness), cognitive (learning difficulties at school), and behavioural and psychological [11]. Globally, the most common sequelae in children with IMD appear to be skin necrosis (10%) and neurological issues (10–12.2%) [12]. In France, a recent retrospective study based on the national health insurance database (SNDS) showed that the neurological sequelae were the most frequent (all ages groups; 5 years follow-up) [13]. However, there are few prospective studies looking at the long-term neuropsychological and developmental consequences of IMD in surviving children and their rate may be underestimated.

Recently, in Australia, the AMEND study started to evaluate the long-term sequelae in young adults [14] but to our knowledge no prospective studies in children have been performed to date. In this context, it seems essential to evaluate in depth the impact of IMD on children development, to have a more reliable estimate of the rate of sequelae and above all to set up an adapted and optimal follow-up of patients surviving IMD in order to establish new management guidelines.

Therefore, the aim of the SEINE study is to assess the long-term physical, neuropsychological, and sensory sequelae of IMD in children and adolescents included in the French paediatric bacterial meningitis surveillance network and to assess the post-traumatic stress in parents. [5, 15].

## Methods

Since 2001, the GPIP/ACTIV (Groupe de Pathologie Infectieuse Pédiatrique and Association Clinique et Thérapeutique Infantile du Val de Marne) set up an active surveillance network to analyse the epidemiological, clinical, biological features and short-term outcome (including sequelae occurring during hospitalisation only) of PF and MM [5, 16]. The methodology was previously described [5, 15, 16]. Briefly, since 2001, 233 pediatric wards and 166 microbiology laboratories reported their IMD among children aged between 0 and 18 years old. The data collection of the French paediatric bacterial meningitis surveillance network was approved by the French National Data Protection Commission (CNIL, no. 913006) and by an ethics committee (CHI Créteil Hospital, France) (NCT04664569). The data collection of the SEINE study was approved on December, 22, 2020 by the French Sud Mediterranean I Person Protection Committee (No. 2020-A00857-32) NCT04685850. Parents who agree to participate in the SEINE study will have the opportunity to discuss the purpose and procedures of the study with the study team. A written consent form will be signed by parents of all the participants before the enrollment in the SEINE study. If one or more physical, neurological, or sensory sequelae are identified during the study, the study team will facilitate referral for follow-up with the appropriate specialist. The results will be disseminated via peer-reviewed publications and conference presentations.

To set up the SEINE study, first, we performed a pilot study to assess whether cases in Paris area were representative of all national cases. To evaluate the feasibility in the Paris area, the criteria used in the pilot study were the number of MM and PF cases in France compared to those in Paris area from 2001 to 2018 by region, age and serogroups.

### Pilot study results

During the study period, 2.543 MM cases with culture confirmed *N*. *meningitidis* and 580 PF cases were reported in the French paediatric bacterial meningitis surveillance network. Most of the cases were caused by serogroup B (n = 1.630; 64.0%) and serogroup C (n = 603; 23.7%).

Cases reported in the Paris area accounted for 14.9% (n = 380) of MM cases and 15.6% (n = 79) of PF cases. Table 1 shows the distribution of IMD by serogroups and age groups in France and in the Paris area during the study period. Children <2 years old accounted for 46.4% of cases. For MM, mean age was 4.4 years ±4.8 (median: 2.4 years) and for PF, mean age was 4.5 years ±4.6 (median: 2.6 years). The age distribution in Paris area was comparable to the age distribution in France for both MM and PF.

**Table 1:** Distribution of invasive meningococcal disease cases by serogroups and age group, France and Paris area, 2001–2018.

Invasive Meningococcal Disease	Age (years)					
	<2 years old		2–11 years old		≥12 years old	
Meningitis serogroups n (%)	France	Paris area	France	Paris area	France	Paris area
	n = 1180 (46.4)	n = 186 (48.8)	n = 1005 (39.5)	n = 158 (41.6)	n = 358 (14.1)	n = 36 (9.5)
**B**	789 (66.8)	111 (59.6)	636 (63.2)	89 (56.3)	205 (57.2)	16 (44.4)
**C**	252 (21.3)	34 (18.2)	247 (24.5)	42 (26.5)	104 (29.0)	10 (27.7)
**W**	47 (3.9)	20 (10.7)	17 (1.6)	5 (3.1)	9 (2.5)	3 (8.3)
**Y**	16 (1.3)	7 (3.7)	16 (1.5)	5 (3.1)	13 (3.6)	3 (8.3)
**Other or unknown serogroup**	75 (6.3)	14 (7.5)	86 (8.5)	17 (10.7)	24 (6.7)	4 (11.1)
**A**	1 (0,08)	0	3 (0.2)	0	2 (0,5)	0
**X**	0	0	0	0	1	0
**Purpura Fulminans**	214 (42.8)	37 (46.8)	221 (43.5)	34 (43.0)	73 (14.4)	8 (10.1)

The pilot study results showed that the distribution of IMD cases by serogroup and age range in the Paris area was similar to the distribution of cases at the national level. According to these preliminary results of the pilot study, the decision was to conduct the SEINE study in the Paris area to assess the long-term sequelae in children surviving MM or PF.

The feasibility of a long-term sequelae assessment study depends on the number of years between the bacterial episode and the follow-up visit. The SEINE Study scientific committee decided to recruit patients admitted to paediatric wards in Paris area from 2010 to 2019 to recruit enough potential cases even if we assume that patients diagnosed between 2010–2019 will have experienced quite a range of time post-diagnosis prior to their first follow-up visit for the SEINE study. Our results will need to account for these delays differences and we will present the results stratified by the delays. Moreover, this follow-up time is in line with the ones found in the literature for study with a similar objective [17, 18].

### SEINE study design

The SEINE study is a multicentre, prospective, non-randomized, interventional study (S2 and S3 Files. SEINE Study Protocol). The primary aim is to assess long-term sequelae (physical, neurological, or sensory) in children and adolescents surviving MM or PF. Secondary aims are (1) to establish a pathway for care, (2) to assess long-term post-traumatic stress among parents of children with IMD and (3) to assess the societal impact of the disease (academic achievement, parents’ activity). 39 paediatric wards in the Paris area will enroll all IMD cases between 2010 and 2019. The inclusion criteria are children aged from birth to 15 years old at time of the study, previously enrolled in the bacterial meningitis surveillance network for IMD. Data extracted from this surveillance network included age, gender, date of disease onset and clinical presentation (MM, PF). Data collected during the study will be recorded in a case report form (CRF) for each patient who agree to participate (S4 File. SEINE Case Report Form).

### Study procedure

**Fig 1:**
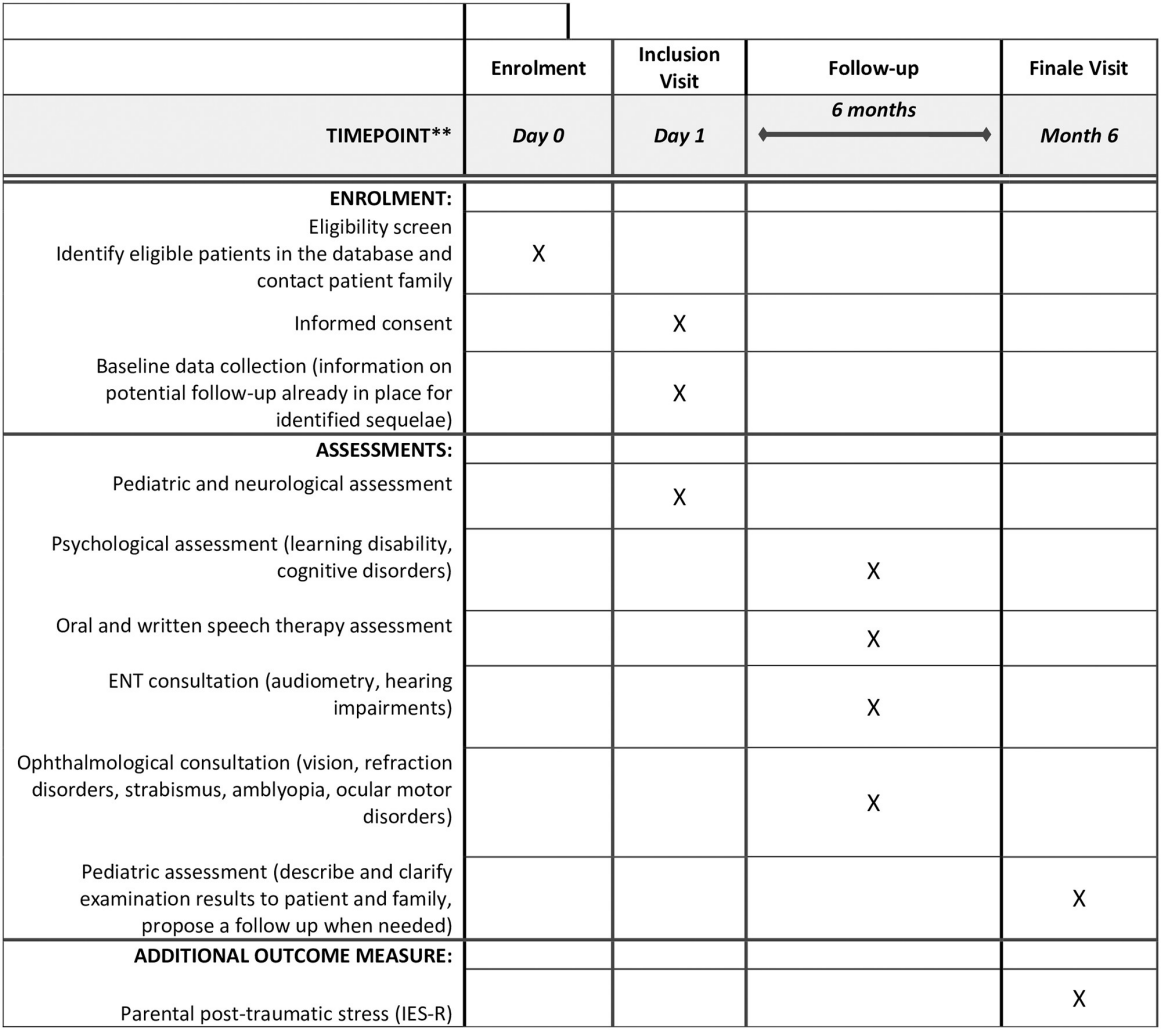
SEINE study procedure flow chart.

The study procedure is presented in Fig 1. Patient enrolment started in May 2021 and will end in May 2023.

### Physical, neurocognitive, and sensory sequelae assessments

#### Inclusion visit

The inclusion visit will be performed by the paediatrician investigator. For all IMD cases, an interview about medical history linked to the MM or PF event and modalities of the sequelae follow-up will be conducted. A general clinical examination followed by orthopaedic, cutaneous, and neurologic examination will be conducted. To seek learning difficulties, patient and/or parents will be questioned about the patient school career. If the patient has already been managed for sequelae, information will be gathered by the investigator centre including reports from consultation, hospitalization, and medical imaging.

#### Neurocognitive sequelae

*Language disorders*. Depending on the child age, a standardized test battery will be administrated by a speech therapist to assess the development of oral and written language. The tests in each battery are randomized and normed (Table 2). One hour and half will be needed to complete the tests.

**Table 2:** Neurocognitive testing.

Domain	Test	Age range	Sub-domain
**Developmental language**	EVALO 2–6	2,3 months to 6,3 years	Oral language, written language, pragmatic skills, functional language architecture
	EXALANG	3 to 15 years	Oral language, written language, and cross-curricular skills.
**General intelligence**	WPPSI IV	2,5 to 5 years	Verbal comprehension, visual-spatial, fluid reasoning, working memory, and processing speed.
	WISC V	≥ 6 years	Verbal comprehension, perceptual reasoning, a working memory and a processing speed.

*Learning and cognitive disorders*. Depending on the child age, an evaluation of learning disorders as well as cognitive disorders will be performed by a neuropsychologist to establish a final score (Table 2). A total score <70 is associated with major cognitive disability; a score between 70 and 129 with low to high intelligence; and ≥ 130 with high intellectual potential. Children for whom the Wechsler Intelligence Scale for Children (WISC) or Wechsler Preschool and Primary Scale of Intelligence (WPPSI) will not be feasible, Vineland scale, a developmental scale based on parental interview will be used. At least one hour and half will be needed to complete the evaluation.

#### Sensory sequelae

*Audiometry and ophthalmic examination*. A pure tone audiometry and an ophthalmic examination will be conducted by a ear, nose, and throat (ENT) specialist and an ophthalmologist, respectively. The audiometry will determine the presence of hearing loss while the visual examination will determine the presence of refractive disorders, strabismus, amblyopia and/or motor ophthalmologic disorders. An orthoptic assessment may be necessary if prescribed by the ophthalmologist. Thirty minutes will be needed for both the audiometry and ophthalmic examination.

#### Parental post-traumatic stress assessment

The Impact of Event Scare Revised (IES-R) questionnaire will be completed online by parents of the included patient. It consists of 22 items assessing parental feelings about their child’s illness and identifies the presence of post-traumatic stress if the total score is ≥ 33.

#### Final visit

This consultation will allow the paediatrician investigator to deliver detailed feedback, including a written report of all evaluations to the patient and his/her family. In case of identified sequelae with no follow-up, the patient will be referred to adapted healthcare professionals. In the same way, the parent will be referred to adapted healthcare professionals in case of identified post-traumatic stress with no follow-up.

### Sample size

Between 2010 and 2019, 169 cases of MM and PF were reported to the French paediatric bacterial meningitis surveillance network. Initial clinical data are already available for these patients, 9 patients died, 36 experienced short-term sequelae, 21 had neurological complications, 8 had circulatory complications and 7 presented with other involvement. After excluding deceased patients (n = 9) and patients lost to follow-up (about 20%, n = 30) and considering an acceptance rate of 80%, 100 patients should be included and benefit from the care pathway established in the SEINE study.

### Statistical analysis

The proportion of children with sequelae, with confidence interval, will be described for the entire cohort recruited, within each age group, and according to the type of sequelae: neurological, orthopaedic, skin, sensory and cognitive. We will present the results stratified by the severity of the presenting condition and by the delay since MM or PF occurred. Qualitative data will be described by their number and percentage, and quantitative data will be described by their mean and standard deviation, and median and interquartile range. Comparison tests could be used to determine the presence of sequelae according to the initial clinical presentation (MM or PF).

### Data management and confidentiality

The investigators will make available the documents and individual data strictly necessary for the monitoring, quality control and auditing of the research according with the current legislative and regulatory laws (Articles L.1121-3 and R.5121-13 of the Public Health Code). Each patient will be given an identification code consisting of a centre number and patient number. The sponsor will ensure that each person who takes part in the research has given his or her written consent for access to individual data and only datra strictly necessary for the quality control of the research. The results are saved on a secure server, approved as "health data".

## Discussion

Few studies have reported the long-term sequelae of MM and PF in children for a minimum of one year and up to 10 years after discharge from the hospital. Our data will help to understand the long-term impact of IMD in young people as well as his/her family and the healthcare system.

The strength of our study is to use a prospective and multicentre approach to estimate and accurately describe long term sequelae. The first limitation of our study is the representativeness of our cohort, by comparing the number of cases reported during the same period in the French National Reference Center for Meningococcus which was higher [19]. However, we will provide qualitative data on sequalae. The second limitation of the SEINE study is the patient enrolment which may be difficult if some of the IMD survivors no longer live in the Paris area. However, we have tried to improve any recruitment bias by providing travel reimbursement for participants.

The SEINE Study has multiple perspectives. First, for patients and parents, the study will allow them to have access to specialists, without incurring costs, enabling them to identify whether their child has sequelae and allowing the corresponding follow-up to be set up to improve the care of the child. In terms of public health, this study should highlight the importance of a structured care pathway for patients with this severe disease with potential late consequences. It will also provide robust data on the impact of IMD on survivors and their families, which can help future cost-effectiveness estimates of children and adolescent vaccination programs.

By providing a more precise estimation of the rate of sequelae in children, providing a thorough clinical examination and offering an adapted follow-up of these children, the SEINE study will help to improve the management of patients surviving IMD.

## Supporting information

S1 File SPIRIT 2013 checklist: Recommended items to address in a clinical trial protocol and related documents. (DOC)

S2 File SEINE Study Protocol: Long-term sequelae of childhood meningitis and meningococcal purpura fulminans in Ile de France: A multidisciplinary approach. 2021 november 22, version C.
(DOCX)

S3 File SEINE Study Protocol French version: Long-term sequelae of childhood meningitis and meningococcal purpura fulminans in Ile de France: a multidisciplinary approach. 2021 november 22, version C.
(DOCX)

S4 File SEINE case report form. (DOCX)
