# Supplementary material for: Long-term impact of invasive meningococcal disease in children: SEINE study protocol
Source: PLoS One. 2022 May 26;17(5):e0268536. doi: 10.1371/journal.pone.0268536 (PMC9135194; doi:10.1371/journal.pone.0268536)
Supplement: S2 File — 2021 november 22, version C. (DOCX) [file pone.0268536.s002.docx]

| Study acronym | **SEINE** (**SE**quella **I**n **NE**isseria) | |
| --- | --- | --- |
| Study title | Long-term sequelae of childhood meningitis and meningococcal purpura fulminans in Ile de France: a multidisciplinary approach | |
| Financial support | SANOFI PASTEUR | Dr Fanchon Laurent  SANOFI LYON Campus  Building A2 — 6^th^ floor  14 Espace Henry Vallée — 69007 Lyon  Laurent.Fanchon@sanofi.com |
| Sponsor | Address | Intercommunal Hospital of Créteil [Centre Hospitalier Intercommunal de Créteil]  40 avenue de Verdun, 94010 Créteil |
|  | Contact | |
|  | Surname, first name | Ms Vanessa Esteves |
|  | Telephone | 01 57 02 20 30 |
|  | Email | [vanessa.esteves@chicreteil.fr](mailto:vanessa.esteves@chicreteil.fr) |
| Coordinating investigator | Surname, first name | Prof. Robert Cohen |
|  | Address | Infant Department  Intercommunal Hospital of Créteil  40 avenue de Verdun, 94010 Créteil |
|  | Email | robert.cohen@activ-france.fr |
| Scientific committee |  | Prof. Robert Cohen, Val de Marne Infant Clinical and Therapeutic Association (ACTIV)  Dr Camille Jung, Paediatrics, Intercommunal Hospital of Créteil (CHIC)  Dr Ines Layouni, CHIC Neonatal Medicine  Dr Corinne Levy, ACTIV  Dr Michaël Levy, Paediatric Intensive Care, Robert Debré Hospital [Hôpital Robert Debré]  Ms Geneviève Monguillot, private practice  Prof. Muhamed-Kheir Taha, National Reference Centre (CNR) for Meningococci and Haemophilus influenzae |
| Associated national reference centre | Surname, first name | Prof. Muhamed-Kheir Taha |
|  | Address | Meningococci CNR  Pasteur Institute  28 rue du Docteur Roux — 75724 Paris CEDEX 15 |
|  | Email | muhamed-kheir.taha@pasteur.fr |
| Methodology/Coordination, analyses | Surname, first name | Dr Corinne Levy and Dr Camille Jung |
|  | Address | Intercommunal Hospital of Créteil (CHI) CRC  40 avenue de Verdun, 94010 Créteil |
|  | Email | corinne.levy@activ-france.fr  [camille.jung@chicreteil.fr](mailto:camille.jung@chicreteil.fr) |
| Data management, Statistics | Surname, first name | Stéphane Bechet |
|  | Address | Val de Marne Infant Clinical and Therapeutic Association [Association Clinique et Thérapeutique Infantile du Val de Marne] (ACTIV)  31, rue Le Corbusier 94300 Créteil |
|  | Email | [stephane.bechet@activ-france.fr](mailto:stephane.bechet@activ-france.fr) |
| Data protection officer | Structure | DPO Consulting |
|  | Email: | [dpo@chicreteil.fr](mailto:dpo@chicreteil.fr) |

**Table of contents**

[1 Protocol signature page 4](#_Toc83200948)

[2 Abbreviations 5](#_Toc83200949)

[3 Project summary 6](#_Toc83200950)

[4 Research rationale and background 9](#_Toc83200951)

[4.1 Research hypothesis 9](#_Toc83200952)

[4.2 Description of the condition 10](#_Toc83200953)

[4.3 Description of the relevant population 11](#_Toc83200954)

[4.4 Benefit(s) 11](#_Toc83200955)

[4.5 Risk(s) and obligation(s) attached to the research 11](#_Toc83200956)

[5 Objectives of the research 11](#_Toc83200957)

[5.1 Primary Objective 11](#_Toc83200958)

[5.2 Secondary Objectives 11](#_Toc83200959)

[6 Subject screening 12](#_Toc83200960)

[6.1 Inclusion criteria 12](#_Toc83200961)

[6.2 Exclusion criteria 12](#_Toc83200962)

[6.3 Enrolment Objective 12](#_Toc83200963)

[7 Research methodology 13](#_Toc83200964)

[7.1 Primary and secondary endpoints 13](#_Toc83200965)

[7.1.1 Primary endpoint 13](#_Toc83200966)

[7.1.2 Secondary endpoint(s) 13](#_Toc83200967)

[8 Performance of the study 13](#_Toc83200968)

[8.1 Patient screening 13](#_Toc83200969)

[8.2 Research procedure 14](#_Toc83200970)

[8.2.1 Diagram of the patient pathway 14](#_Toc83200971)

[14](#_Toc83200972)

[8.2.2 Visit details 14](#_Toc83200973)

[8.2.3 Visit schedule 15](#_Toc83200974)

[8.2.4 Details of the study assessments and evaluations 16](#_Toc83200975)

[8.3 Research schedule: 17](#_Toc83200976)

[9 Safety assessment 18](#_Toc83200977)

[9.1 Monitoring committee 18](#_Toc83200978)

[10 Description of the rules for permanent or temporary discontinuation 18](#_Toc83200979)

[10.1 Discontinuation of a person’s participation in the study 18](#_Toc83200980)

[10.2 Discontinuation of all or part of the research by the sponsor. 18](#_Toc83200981)

[11 Data management 19](#_Toc83200982)

[11.1 Data collected 19](#_Toc83200983)

[11.2 Right of access to the data 19](#_Toc83200984)

[11.3 Confidentiality 19](#_Toc83200985)

[11.4 Archiving 20](#_Toc83200986)

[11.5 Control and Quality Assurance 20](#_Toc83200987)

[12 Statistical aspects 20](#_Toc83200988)

[12.1 Assessment of the number of subjects to be recruited 20](#_Toc83200989)

[12.2 Analyses 20](#_Toc83200990)

[13 Ethical and legal aspects 21](#_Toc83200991)

[13.1 Legal obligations 21](#_Toc83200992)

[13.2 Sponsor 21](#_Toc83200993)

[13.3 EC submission 21](#_Toc83200994)

[13.4 Substantial amendments 21](#_Toc83200995)

[13.5 Computerised data — French Data Protection Authority (CNIL) 21](#_Toc83200996)

[13.6 Insurance 21](#_Toc83200997)

[14 Publication rules 22](#_Toc83200998)

[14.1 Scientific communications 22](#_Toc83200999)

[14.2 Communication of the results to the participants 22](#_Toc83201000)

[14.3 Data transfer 22](#_Toc83201001)

[15 Bibliographic references 23](#_Toc83201002)

[16 ADDENDUM 24](#_Toc83201003)

[16.1 List of participating sites 24](#_Toc83201004)

# Protocol signature page

| Study acronym | SEINE (***SE****quella* ***I****n* ***NE****isseria*) |
| --- | --- |
| Study title | Long-term sequelae of childhood meningitis and meningococcal purpura fulminans in Ile de France: a multidisciplinary approach |
| ID-RCB Number | **2020-A00857-32** |

| Sponsor | | |
| --- | --- | --- |
| Intercommunal Hospital of Créteil  40, avenue de Verdun  94010 Créteil CEDEX | Name | Catherine Vauconsant  Managing Director |
|  | Créteil, on |  |
|  | Signature: |  |

| Coordinating investigator | | |
| --- | --- | --- |
| Intercommunal Hospital of Créteil  40 avenue de Verdun,  94010 Créteil CEDEX | Name | Robert Cohen |
|  | Créteil, on |  |
|  | Signature: |  |

# Abbreviations

| ACTIV | *Association clinique thérapeutique infantile du Val de Marne* [Val de Marne Infant Clinical and Therapeutic Association] |
| --- | --- |
| CRA | Clinical Research Associate |
| CHIC | *Centre Hospitalier Intercommunal de Créteil* [Intercommunal Hospital of Créteil] |
| CRC | Clinical Research Centre |
| PI | Principal Investigator |
| RIPH | *Recherche interventionnelle sur la personne humaine* [Interventional Research on Humans] |
| CST | Clinical Study Technician |
| GPIP | *Groupe de Pathologie Infectieuse Pédiatrique* [French Paediatric Infectious Disease Group] |

# Project summary

| **General characteristics** | | |
| --- | --- | --- |
| Study acronym | SEINE | |
| ID-RCB Number | **2020-A00857-32** | |
| Clinical Trials Number | NCT04685850 | |
| Sponsor | Intercommunal Hospital of Créteil | |
| Regulatory | | |
| Study type | Study with minimal risks and obligations involving human subjects (RIPH2) | |
| Number of sites | 56 hospital departments in Ile de France | |
| Methodology | A multi-centre, prospective, non-randomised study of patients who had an invasive meningococcal infection (IMI) such as meningitis or meningococcal purpura fulminans diagnosed in a hospital paediatric department in Ile de France between 2010 and 2019. | |
| **Research design** | | |
| Population involved | Children aged 1 to 15 years who had meningitis or meningococcal purpura fulminans between 2010 and 2019 | |
| Rationale and background | The occurrence of an IMI, mainly meningitis, is of concern not only to healthcare professionals but also to the general population, in whom this condition justifiably remains a major worry. Thanks to the monitoring networks supported by the French National Public Health Agency [Santé Publique France] (Meningococci CRN, Epibac network), we have reliable data on incidence and mortality in France. The same is not true for the sequelae. Due to its methodology, the French Observatory of Bacterial Meningitis in Children [Observatoire des Méningites Bactériennes de l’Enfant] established in 2001 by the French Paediatric Infectious Disease Group (GPIP) allows for a short-term approach to sequelae. In contrast, the assessment of more long-term sequelae, on the clinical level and on the intellectual, sensory and learning development level, requires patients to be followed-up for a minimum of one year after the onset of the disease.  The French Observatory of Bacterial Meningitis in Children identified 7,624 cases of meningitis and purpura fulminans from all bacteria between 2001 and 2019 from 233 participating paediatric departments. In Ile de France, 56 general paediatric, neonatal medicine and paediatric intensive care departments made up 22% of this cohort. If the period from 2010 to 2019 is considered, 169 cases of meningitis and meningococcal purpura fulminans were identified (15 to 20 cases per year); nine patients died, 36 experienced short-term sequelae, 21 had neurological complications, eight had circulatory complications and seven presented with other involvement. A recent survey carried out with the relevant departments in Ile de France showed that the patients were, on average, seen again one year after the episode, and that some of them had sequelae (intellectual or physical) which were not initially identified at the time the patient was discharged from hospital.  Thus, by selecting only the Ile de France region (representative of the rest of France), the observational data could be used to implement a prospective study which would allow for a more specific approach to the long-term sequelae of childhood meningitis and meningococcal purpura fulminans. This would specifically include clinical, psychological and psychomotor assessments and a speech assessment. In addition, a survey addressed to the parents should help to assess their experience with the disease, to identify post-traumatic stress, and to review the care pathway which had (or had not) been implemented since their child was discharged from hospital. The perception of the relatives regarding the consequences of these serious conditions, such as amputations and their medical and logistical care, might thus also be described. | |
| Expected results | Few studies have reported on the long-term sequelae of childhood IMIs, such as meningitis and purpura fulminans, at least one year after, and up to a maximum of ten years after, their discharge from hospital. In terms of public health, this study should highlight the importance of a “flagged“ care pathway in these serious conditions which sometimes have delayed consequences. | |
| Calculation of the number of patients | Between 2010 and 2019, 169 cases of meningitis and meningococcal purpura fulminans were declared in the French National Observatory of Bacterial Meningitis in Children. After having excluded deceased patients (n = 9) and patients who have been lost to follow-up (approximately 20%, n = 30), and with an envisaged acceptance rate of 80%, 100 patients should be enrolled and follow the care pathway established as part of the study. | |
| Primary objective | To assess the long-term (between one and ten years) physical, neurological or sensory sequelae of meningitis or meningococcal purpura fulminans | |
| Primary endpoint | - Number of children with at least one neurological, orthopaedic or sensory sequela | |
| Secondary objectives | - To establish a care pathway extending beyond one year of follow-up - Assessment of parental post-traumatic stress - To assess the social impact of the disease: education of the children; activity of the parents | |
| Secondary endpoints | - Number of children who underwent a “flagged“ care pathway with the establishment of a score based on the consultations performed - Number of children with severe neurological sequelae: hemiplegia etc. - Number of children with orthopaedic complications (amputations etc.) - Number of children with sensory disorders - Number of children with skin sequelae - Number of children with learning disabilities (with no identified neurological sequelae) - Number of children with cognitive or psychological impairments identified on the WPPSI, WISC V or VINELAND - Care pathway followed by the children: number and type of follow-up consultations (Specialised paediatric consultations or not; ENT and speech therapy assessment/follow-up; neurodevelopmental assessment) - Parental post-traumatic stress assessment via the IES-R questionnaire | |
| Inclusion criteria | - Infants and children 1 to 15 years of age - Having had meningitis or meningococcal purpura fulminans included in the observatory between 2010 and 2019 - Those with parental authority have read and understood the information letter and their express consent was obtained - Patient affiliated with a social security scheme (Social Security or French Universal Medical Coverage) | |
| Exclusion criteria | - Refusal of either parent | |
| Performance of the study | | |
| Patient pathway | 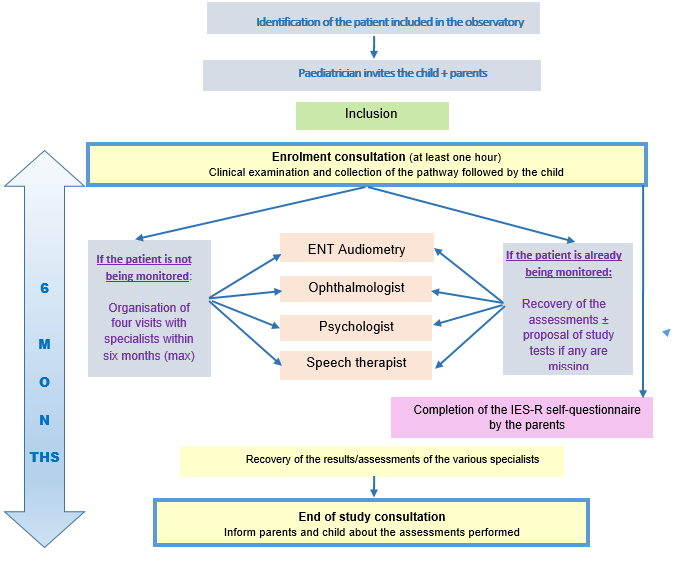 | |
| Study schedule | Planned start date of the study | Q4 2020 |
|  | Planned enrolment end date | Q2 2023 |
|  | Duration of the enrolment period | 2 years |
|  | Duration of patient follow-up in the study | 1 year |
|  |  | |
| Publications/Communications | Publication in a peer-reviewed medical journal  Presentation of the study (oral or poster) at a conference | |

# Research rationale and background

Research hypothesis

The occurrence of invasive meningococcal infections (IMI), mainly meningitis, is of concern not only to healthcare professionals but also to the general population, in whom this condition justifiably remains a major worry. Thanks to the monitoring networks supported by the French National Public Health Agency (Meningococci CRN, Epibac network), we have reliable data on incidence and mortality in France. The same is not true for the sequelae. Due to its methodology, the French Observatory of Bacterial Meningitis in Children established in 2001 by the French Paediatric Infectious Disease Group (GPIP) allows for a short-term approach to sequelae. In contrast, the assessment of more long-term sequelae, on the clinical level and on the intellectual, sensory and learning development level, requires patients to be followed-up for a minimum of one year after the onset of the disease.

The French Observatory of Bacterial Meningitis in Children identified 7,624 cases of meningitis and purpura fulminans from all bacteria between 2001 and 2019 from 233 participating paediatric departments. In Ile de France, 56 general paediatric, neonatal medicine and paediatric intensive care departments made up 22% of this cohort. If the period from 2010 to 2019 is considered, 169 cases of meningitis and meningococcal purpura fulminans were identified (15 to 20 cases per year); nine patients died, 36 experienced short-term sequelae, 21 had neurological complications, eight had circulatory complications and seven presented with other involvement. A recent survey carried out with the relevant departments in Ile de France showed that the patients were seen, on average, one year after the episode, and that some of them had sequelae (intellectual or physical) which were not initially identified at the time the patient was discharged from hospital.

Thus, by selecting only the Ile de France region (considered as being representative of the rest of France), the observational data could be used to implement a prospective study which would allow for a more specific approach to the long-term sequelae of childhood meningitis and meningococcal purpura fulminans. This would specifically include clinical, psychological and psychomotor assessments and a speech assessment. In addition, a survey addressed to the parents should help to assess their experience with the disease, to identify post-traumatic stress and to review the care pathway which had (or had not) been implemented since their child was discharged from hospital. This study will thus assess the consequences of these serious conditions with their medical and logistical care as well as the impact that they have on their relatives.

This study’s primary objective is to assess the long-term sequelae of childhood IMIs such as meningitis and purpura fulminans using a multidisciplinary psychological, intellectual, sensory and clinical approach. The secondary objective is to assess the social consequences and the perception of relatives regarding this serious condition, so that the true burden of the disease may be correctly measured by taking into account parameters which have not been considered to date.

Description of the condition

IMIs include all serious infections related to meningococci or *Neisseria meningitidis* (Nm). An invasive infection is defined by the isolation of a bacteria in a space which is normally sterile (blood, cerebrospinal fluid etc.) and the two main infections are meningitis and septicaemia (1). They may progress to septic shock including to purpura fulminans, the most lethal form of the disease. This is characterised clinically by extensive and rapidly necrotic, ecchymotic purpura combined with circulatory failure (2).

Nm is a bacterium which is specific to humans. In approximately 10% of the general population, it colonises the mucous membrane of the upper respiratory tract of humans without causing an IMI (3). In rare cases, some strains of Nm, known as hyperinvasive strains (4), can cross the respiratory epithelium and invade and multiply in the bloodstream, causing an IMI.

In industrialised countries (Europe, America and Oceania), the annual incidence varies from 0.7 to 2 cases per 100,000 inhabitants and mainly occurs in sporadic cases, with a predominance of strains of serogroups B and C. Thus, the incidence of IMI in France remains low (close to the average rate of 1 in 100,000 observed in Europe) but is higher in infants under the age of one (8.9 in 100,000 in 2015) and young adults aged 18–20 (1.5 in 100,000 in 2015) (5). The majority of cases occur sporadically and mainly relate to the B serogroups, which account for an average of 62% of cases (5).

It is a severe condition, as the mortality rate is from 8 to 10%, and it accounts for 50 to 60 deaths per year (5). It may reach much higher rates in the case of purpura fulminans (6). As regards the sequelae, immediate sequelae are well known but there are few studies which focus on the long-term sequelae and on the cognitive development of children. According to studies, sequelae secondary to an IMI at the end of hospitalisation were reported in 10 to 40% of cases (7–10). These are usually cutaneous (skin necrosis with more or less extensive loss of skin which may require a skin graft), orthopaedic (amputations), neurological (focal neurological deficits, epilepsy, spasticity, deafness), cognitive (learning disabilities), behavioural and psychological. The most common sequelae in children who have had an IMI appear to be skin necrosis (10%) and all types of neurological problems (10 to 12.2%) (11).

However, there are few prospective studies which focus on the later neuropsychological and developmental consequences for these children, and the rate of delayed sequelae is possibly being underestimated at the moment. The ongoing AMEND study is assessing these long-term sequelae in young adults (12), but a prospective study on children is also necessary. In fact, the latter seems essential to thoroughly assess the impact these IMIs may have on development, to obtain a more reliable estimate of the rate of sequelae and, above all, to be able to implement appropriate monitoring of these children.

Description of the relevant population

This study will involve children from 0 to 15 years of age who had meningitis or meningococcal purpura fulminans diagnosed in a hospital paediatric department in Ile de France between 2010 and 2019.

Benefit(s)

Few studies have reported on the long-term sequelae of childhood meningitis and meningococcal purpura fulminans at least one year after, and up to a maximum of 10 years after, their discharge from hospital. In terms of public health, this study should highlight the importance of a “flagged“ care pathway in these serious conditions which sometimes have delayed consequences.

This study will allow parents to have access to specialists, at no cost, allowing them to identify whether their child has experienced sequelae and allowing the implementation of a corresponding follow-up to improve the management of the child.

Risk(s) and obligation(s) attached to the research

The study does not involve any risks. The only obligation is the need to attend consultations with various specialists.

# Objectives of the research

Primary Objective

To assess the long-term (between one and ten years) physical, neurological or sensory sequelae from meningitis or meningococcal purpura fulminans.

Secondary Objectives

- Establish a course of care
- To assess medium/long-term post-traumatic stress on parents
- To assess the social impact of the disease: education of the children; activity of the parents

# Subject screening

Inclusion criteria

- Infants and children 1 to 15 years of age;
- Who had meningitis or meningococcal purpura fulminans included in the observatory between 2010 and 2019;
- One of the individuals with parental authority has read and understood the information letter and their express consent has been obtained;
- Patient affiliated with a social security scheme (Social Security or Universal Medical Coverage).

Exclusion criteria

- Refusal of either parent.

Enrolment Objective

Approximately 100 patients are expected to be enrolled in the study.

# Research methodology

This is an interventional study with minimal risks and obligations involving human subjects (RiPH2).

It is a multi-centre, prospective, non-randomised study.

Primary and secondary endpoints

### Primary endpoint

- Number of children with at least one neurological, skin, orthopaedic or sensory sequela.

### Secondary endpoint(s)

- Number of children who underwent a “flagged“ care pathway with the establishment of a score based on the consultations performed.
- Number of children with some severe neurological sequelae: hemiplegia etc.
- Number of children with orthopaedic complications (amputations etc.).
- Number of children with sensory disorders.
- Number of children with skin sequelae.
- Number of children with learning disabilities (with no identified neurological sequelae).
- Number of children with cognitive or psychological impairments identified on the WPPSI, WISC V or VINELAND. Care pathway followed by the children: number and type of follow-up consultations; Specialised paediatric consultations or not; ENT and speech therapy assessment/follow-up; neuro-developmental assessment.
- Parental post-traumatic stress assessment via the IES-R questionnaire.

# Performance of the study

Patient screening

The study will be offered to parents of children reported in the French National Observatory of Bacterial Meningitis in Children who are not deceased and have not been lost to follow-up. If the parents agree to participate in the protocol, and after the informed consent form has been obtained, the children will be enrolled.

Research procedure

### Diagram of the patient pathway

##

**Identification of the patient included in the observatory**

**Paediatrician invites the child + parents**

Inclusion

**6**

**6**

**M**

**O**

**N**

**THS**

**Enrolment consultation** (at least one hour)

Clinical examination and collection of the pathway followed by the child

**If the patient is already being monitored:**

Recovery of the assessments ± proposal of study tests if any are missing

**If the patient is not being monitored**:

Organisation of four visits with specialists within six months (max)

ENT Audiometry

Ophthalmologist

Psychologist

Tests according to age

Speech therapist

Tests according to age

Completion of the IES-R self-questionnaire

by the parents

Recovery of the results/assessments of the various specialists

**End of study consultation**

Inform parents and child about the assessments performed

### Visit details

- **Enrolment visit**

This enrolment consultation with the investigating paediatrician will last at least one hour and will include:

- An interview regarding the meningitis-related to the meningitis (skin sequelae, hospitalisations since the meningitis) and any follow-ups.
- General clinical, orthopaedic and skin examinations.
- Neurological examination.
- The follow-up information will be collected by the recruiting site: consultation/hospitalisation assessments and imaging reports (brain MRI, if performed).
- Child‘s school history, screening for learning disability.
- **Specialised assessments, a maximum of four**

The purpose of the specialised assessments is to assess possible sensory sequelae:

- ENT assessment and audiometry.
- Vision assessed by an ophthalmologist.
- Screening for oral and written speech disorders by a speech therapist.
- The child’s intellectual development will be assessed using the WISC IV or V performed by a psychologist. If the WISC cannot be performed (too significant a retardation), the Vineland scale will be used to assess the child’s development.

If the child is not being monitored by one or more specialists, a list of professionals will be provided to the child’s parents for an appointment within six months of the enrolment visit.

If the child is being monitored, the findings from previous assessments will be collected and, if needed, completed by the professional monitoring the child.

- **End of study visit with the referring paediatrician**

This visit will allow the hospital physician monitoring the child to review the situation with the parents and provide feedback on the specialised assessments and possible management.

*Assessment of parental post-traumatic stress*:

Parental post-traumatic stress will be assessed through the Impact of Event Scale-Revisited (IES-R) questionnaire. The parents will be able to complete this questionnaire online through a dedicated site.

### Visit schedule

| Procedure/examination | Inclusion | 6-month period | End of study visit |
| --- | --- | --- | --- |
| Signing of the informed consent form | (*)✓ |  |  |
| Paediatric/neurological assessment | ✓ |  | (*)✓ |
| Psychological assessment |  | ✓ |  |
| Speech therapy assessment |  | ✓ |  |
| ENT consultation (audiometry) |  | ✓ |  |
| Ophthalmology consultation (vision) |  | ✓ |  |
| Assessment of parental post-traumatic stress |  |  | (*)✓ |

*(*) These procedures/examinations are performed within the context of the research and not performed during routine clinical practice*

### Details of the study assessments and evaluations

If the child’s assessments and/or examinations were performed more than one year previously, they will be repeated.

- **Paediatric assessment**

Detailed interview, general clinical, skin, orthopaedic and neurological examinations. Medical and education history, type of follow-up implemented.

- **Speech therapy assessment**

Various tests will be proposed to the child according to their age

- Under four years of age: **EVALO 2-6,** a battery of oral language development tests for children from 2 years and 3 months to 6 years and 3 months.

There are two versions of this: the “small“ version for up to 4 years and 3 months and the “large“ version for older children. Using a range of tests grouped by subject, it explores various skills separately (linguistic skills, pragmatic skills, functional architecture of language), which test both expression and comprehension.

- Above four years of age: **EXALANG battery of digital tests** on the HAPPYNEURON platform.

This is actually composed of five batteries of tests, which include an examination of oral language, written language and cross-sectional skills for children from 3 to 20 years.

It is divided into age groups: 3–6 years, 5–8 years, 8–11 years and 11–15 years.

Each battery of tests is randomised, so that the desired tests and the order in which they are used can be selected (there is no obligation to use an entire set). The tests are standardised.

This tool allows the data analysed to be coupled with the clinical observation. The results are stored on a secure and certified “health data“ server.

Assessment duration: 1.5 hrs.

- **Developmental/psychological assessment**

Various tests will be proposed to the child according to their age with a learning disability assessment as well as an assessment of cognitive disorders in order to establish a final score.

- - **WPPSI IV** (Between 2.5 and 6 years of age):

IQ test in several dimensions: verbal, visual-spatial comprehension, fluid reasoning, working memory and processing speed.

- - **WISC V** (From 6 years of age):

IQ test including a verbal comprehension index, visual-spatial index, fluid reasoning index, working memory index and a processing speed index.

A total score of ≤70 is associated with a mental retardation; a score between 70 and 130 is low to above-average intelligence; and above 130 is higher intellectual potential.

- - The **Vineland Scale** (behavioural assessment) can be performed for all ages and will be proposed for children for whom the WISC or WPPSI cannot be performed. Development will be measured on a development scale based on an interview with the parents.

Developmental age and standard deviation compared with chronological age.

Assessment duration: at least 1.5 hrs.

- **Ophthalmology assessment**

The examination will determine the presence of refraction disorders, strabismus,

amblyopia and/or ocular motor disorders.

An orthoptic assessment may potentially be necessary if it is prescribed by the ophthalmologist.

Assessment duration: 20 mins.

- **ENT assessment**

Performance of an audiogram (to be repeated if the audiogram was done more than one year previously).

Assessment duration: 10 mins.

- **Assessment of parental post-traumatic stress**

The IES-R is a validated, 22-question, self-administered questionnaire in French. It assesses the parent’s experience of their child’s disease and identifies the presence of post-traumatic stress (total score ≥33).

This survey will be offered online.

Research schedule:

| Enrolment start date: | Q4 2020 |
| --- | --- |
| Enrolment duration: | 24 months |
| Patient follow-up end date: | Q4 2023 |
| Date of publication of results: | Q2 2024 |
| Duration of subject participation: | 12 months |

# Safety assessment

According to French Legislation, the surveillance of studies with minimal risks and obligations is the same as that performed as part of normal care. Adverse events will be reported to the corresponding organisation’s pharmacovigilance, medical device vigilance or biovigilance. Note that no drugs are being tested in this study. Monitoring of the expected adverse effects will be specifically performed for the study.

Monitoring committee

Given the nature of the study (interventional study with minimal risks and obligations), no monitoring committee is envisaged.

# Description of the rules for permanent or temporary discontinuation

Discontinuation of a person‘s participation in the study

Patients enrolled in the study may request to withdraw from the study at any time and for any reason.

The investigator may temporarily or permanently discontinue a patient’s participation in the study for any reason which would be in the patient’s best interest.

A patient’s withdrawal from the study will not change the patient’s usual management.

If the patient is lost to follow-up, the investigator will do everything possible to contact the patient. In the event of early withdrawal from the study, the investigator must document the reasons as completely as possible in the medical record.

The data collected on patients who are lost to follow-up or have withdrawn from the study will be used at the time of the analyses.

If consent is withdrawn, and in compliance with regulations, the data will be used up to the date the consent was withdrawn.

Discontinuation of all or part of the research by the sponsor.

Unexpected events, due to which the study objectives are not likely to be met, may lead the sponsor to prematurely discontinue the study.

The sponsor reserves the right to discontinue the study at any time if the sponsor decides that the enrolment objectives have not been reached.

If the study is discontinued early, the information will be sent to the EC by the sponsor within 15 days.

# Data management

Data collected

The following data will be collected for each enrolled patient:

- Medical history
- Clinical examination
- Education
- Audiometry and visual examination results
- Speech therapy and psychological assessment results
- Educational background, social services management
- Results of the parental post-traumatic stress questionnaire

All data will first be collected through a paper questionnaire. The data will then be entered into an electronic case report form.

Right of access to the data

The investigators will provide the documents and individual data which are strictly necessary for follow-up, quality control and research audit to those who have access to these documents in accordance with current legislative and regulatory provisions (Articles L.1121-3 and R.5121-13 of the French Public Health Code).

Confidentiality

In accordance with the current legislative provisions (articles L.1121-3 and R.5121-13 of the French Public Health Code), those with direct access to the source data will take all necessary precautions to ensure the confidentiality of the information related to the research and to those involved, in particular as regards their identity and the findings. These individuals, and likewise the investigators themselves, are subject to professional secrecy.

Each patient will be assigned an identification code consisting of a site number and a patient number (3 digits).

The sponsor will ensure that each person involved in the research has given their approval in writing for access to individual data concerning them which is strictly necessary to check the quality of the research.

Archiving

The following documents regarding research are archived by the sponsor in compliance with Good Clinical Practices and current regulations for a period of 15 years after the end of research.

Control and Quality Assurance

Prior to the analysis of the data, screening for outliers, inconsistencies and missing data will be performed. The investigator will be contacted again for any corrections. There will be no on-site data monitoring.

An audit may be carried out at any time by individuals authorised by the sponsor and independent of the research managers. The purpose of the audit is to ensure the quality of the research, the validity of the results and that the applicable laws and regulations are respected.

The investigators agree to comply with the sponsor’s requirements and with the competent authority as regards any research audit or inspection.

# Statistical aspects

Assessment of the number of subjects to be recruited

Between 2010 and 2019, 169 cases of meningitis and meningococcal purpura fulminans were declared in the French National Observatory of Bacterial Meningitis in Children. The initial clinical data for these patients are already known. After having excluded deceased patients (n = 9) and patients who have been lost to follow-up (approximately 20%, n = 30), and with an envisaged acceptance rate of 80%, 100 patients should be enrolled and follow the care pathway established as part of the study.

Analyses

The proportion and confidence interval of children with sequelae will be described for the entire recruited cohort within each age group and according to the type of sequelae: neurological, orthopaedic, skin, sensory or developmental. The qualitative data will be described by their number and percentage. The quantitative data will be described by their median and standard deviation. Kaplan Meier survival curves will be drawn to determine the presence of sequelae at various times after meningitis. Comparison tests will be performed to determine the presence of sequelae according to their severity or the initial onset.

# Ethical and legal aspects

Legal obligations

The sponsor and the individuals who direct and monitor the research undertake to ensure that this research is performed in accordance with law No. 2012-300 of 5 March 2012 relating to public health policy and current regulatory provisions. (Articles L1121-1, paragraph 2 and R1121-3 of the French Public Health Code).

The research is conducted in accordance with this protocol.

Sponsor

The Intercommunal Hospital of Créteil (CHIC) is the sponsor of this research.

EC submission

This clinical trial received a favourable opinion from the Sud Méditerranée 1 [South Mediterranean 1] EC on 22/12/2020.

Substantial amendments

Requests for substantial amendments will be sent to the sponsor for the opinion of the relevant ethics committee.

The amended protocol should be the subject of an updated version and should be dated.

The information sheets and patient consent forms should be amended, if necessary.

Computerised data — French Data Protection Authority (CNIL)

Data processing will be performed under the confidentiality conditions defined by deliberation No. 2018-153 of 3 May 2018 amending the baseline methodology for personal data processing performed as part of research involving human subjects (MR-001).

Insurance

The Sponsor, CHI Créteil, declares that they have taken out an insurance policy with SHAM, policy No. 102.760, which covers the financial consequences of their civil liability resulting from the application of article L 1121-10 of the French Public Health Code in accordance with the clauses provided in the contract and within the set amounts.

# Publication rules

Scientific communications

Any written or oral communication of the research results must receive prior approval from the sponsor and the coordinating investigator.

The main results of this study will be published in an international peer-reviewed journal in English. The members of the scientific committee and the investigators will be mentioned on original and associated publications.

SANOFI’s financial support for this trial will be mentioned.

Communication of the results to the participants

Participants shall be informed, on demand, of the overall findings of the research in compliance with law No. 2002-303 of 4 March 2002.

Data transfer

The Créteil CHI is the owner of the data.

The conditions under which all or part of the research database are transferred are decided by the research sponsor and are subject to a written contract.

# Bibliographic references

1. Nadel S, Ninis N. Invasive Meningococcal Disease in the Vaccine Era. Front Pediatr. 2018;6:321.

2. Chalmers E, Cooper P, Forman K, Grimley C, Khair K, Minford A, et al. Purpura fulminans: recognition, diagnosis and management. Arch Dis Child. Nov 2011;96(11):1,066–71.

3. Yazdankhah SP, Caugant DA. Neisseria meningitidis: an overview of the carriage state. J Med Microbiol. Sept 2004;53(Pt 9):821–32.

4. Zarantonelli ML, Lancellotti M, Deghmane AE, Giorgini D, Hong E, Ruckly C, et al. Hyperinvasive genotypes of Neisseria meningitidis in France. Clin Microbiol Infect Off Publ Eur Soc Clin Microbiol Infect Dis. May 2008;14(5):467–72.

5. Parent du Chatelet I, Deghmane AE, Antona D, Hong E, Fonteneau L, Taha MK, et al. Characteristics and changes in invasive meningococcal disease epidemiology in France, 2006–2015. J Infect. June 2017;74(6):564–74.

6. Campsall PA, Laupland KB, Niven DJ. Severe meningococcal infection: a review of epidemiology, diagnosis, and management. Crit Care Clin. July 2013;29(3):393–409.

7. Pace D, Pollard AJ. Meningococcal disease: clinical presentation and sequelae. Vaccine. 30 May 2012;30 Suppl 2:B3–9.

8. Ó Maoldomhnaigh C, Drew RJ, Gavin P, Cafferkey M, Butler KM. Invasive meningococcal disease in children in Ireland, 2001–2011. Arch Dis Child. Dec 2016;101(12):1,125–9.

9. Huang L, Heuer OD, Janßen S, Häckl D, Schmedt N. Clinical and economic burden of invasive meningococcal disease: Evidence from a large German claims database. PloS One. 2020;15(1):e0228020.

10. Stein-Zamir C, Shoob H, Sokolov I, Kunbar A, Abramson N, Zimmerman D. The clinical features and long-term sequelae of invasive meningococcal disease in children. Pediatr Infect Dis J. July 2014;33(7):777–9.

11. Wang B, Clarke M, Thomas N, Howell S, Afzali HHA, Marshall H. The clinical burden and predictors of sequelae following invasive meningococcal disease in Australian children. Pediatr Infect Dis J. March 2014;33(3):316–8.

12. Marshall H, McMillan M, Wang B, Booy R, Afzali H, Buttery J, et al. AMEND study protocol: a case-control study to assess the long-term impact of invasive meningococcal disease in Australian adolescents and young adults. BMJ Open. 29 2019;9(12):e032583.

# ADDENDUM

List of participating sites

| No. | Name of the site | Department | Address of the site | Surname of the principal investigator | First name of the principal investigator |
| --- | --- | --- | --- | --- | --- |
| 1 | Robert Debré Hospital | General Paediatrics | 48 Bd Sérurier 75019 Paris | FAYE | Albert |
| 2 | North-Essonne Hospital Group [Groupe Hospitalier Nord Essonne] | Paediatrics | 159 rue du président F. Mitterrand 91345 Longjumeau | GASCHIGNARD | Jean |
| 3 | Paris University Hospital Antoine Béclère South Site [CHU Paris Sud site Antoine Béclère] | General Paediatrics | 157 rue de la porte de trivaux 92140 Clamart | MILCENT | Karen |
| 4 | Argenteuil Hospital [CH Argenteuil] | General Paediatrics | 69 Rue du Lieutenant-Colonel Prudhon, 95107 Argenteuil | BENSAID | Philippe |
| 5 | F Quesnay Hospital [CH F Quesnay] | Paediatrics | 2 boulevard Sully 78200 Mantes la Jolie | PELLEGRINO | Béatrice |
| 6 | Eastern Ile Grand Hospital (GHEF) Meaux site | Paediatrics | 6–8 rue Saint Fiacre 77100 Meaux | VIGNAUD | Olivier |
| 7 | Necker Children’s Hospital [Hôpital Necker Enfants Malades] | Paediatric Metabolic Diseases | 149 rue de Sèvres  75015 Paris | PICHARD | Samia |
| 8 | Créteil CHI | Paediatrics | 40 avenue de Verdun 94010 Créteil | MADHI | Fouad |
