# Supplementary material for: Long-term impact of invasive meningococcal disease in children: SEINE study protocol
Source: PLoS One. 2022 May 26;17(5):e0268536. doi: 10.1371/journal.pone.0268536 (PMC9135194; doi:10.1371/journal.pone.0268536)
Supplement: S3 File — 2021 november 22, version C. (DOCX) [file pone.0268536.s003.docx]

| Acronyme de l’étude | **SEINE** (**SE**quella **I**n **NE**isseria) | |
| --- | --- | --- |
| Titre de l’étude | Séquelles à long terme des méningites et des purpura fulminans à méningocoques de l’Enfant en Ile de France : une approche multidisciplinaire | |
| Soutien financier | SANOFI PASTEUR | Dr Fanchon Laurent  Campus SANOFI LYON  Bâtiment A2 – 6^ème^ étage  14 Espace Henry Vallée - 69007 Lyon  Laurent.Fanchon@sanofi.com |
| Promoteur | Adresse | Centre Hospitalier Intercommunal de Créteil  40 avenue de Verdun, 94010 Créteil |
|  | Contact | |
|  | Nom, prénom | Mme Esteves Vanessa |
|  | Téléphone | 01 57 02 20 30 |
|  | Mail | [vanessa.esteves@chicreteil.fr](mailto:vanessa.esteves@chicreteil.fr) |
| Investigateur coordinateur | Nom, prénom | Pr Cohen Robert |
|  | Adresse | Service des Petits Nourrissons  Centre Hospitalier Intercommunal de Créteil  40 avenue de Verdun, 94010 Créteil |
|  | Mail | robert.cohen@activ-france.fr |
| Comité scientifique |  | Pr Cohen Robert, ACTIV  Dr Jung Camille, pédiatrie CHIC  Dr Layouni Ines, néonatalogie CHIC  Dr Levy Corinne, ACTIV  Dr Levy Michaël, réanimation pédiatrique, hôpital Robert Debré  Mme Monguillot Geneviève, cabinet libéral  Pr Taha Muhamed-Kheir, CNR des Méningocoques et Haemophilus influenzae |
| Centre de référence national associé | Nom, prénom | Pr Taha Muhamed-Kheir |
|  | Adresse | CNR Méningocoque  Institut Pasteur  28 rue du Docteur Roux - 75724 Paris Cedex 15 |
|  | Mail | muhamed-kheir.taha@pasteur.fr |
| Méthodologie/ Coordination, analyses | Nom, prénom | Dr Levy Corinne et Dr Jung Camille |
|  | Adresse | CRC CHI Créteil  40 Avenue de Verdun, 94010 Créteil |
|  | Mail | corinne.levy@activ-france.fr  [camille.jung@chicreteil.fr](mailto:camille.jung@chicreteil.fr) |
| Data management, Statistiques | Nom, prénom | Bechet Stéphane |
|  | Adresse | Association Clinique et Thérapeutique Infantile du Val de Marne (ACTIV)  31, rue Le Corbusier 94300 Créteil |
|  | Mail | [stephane.bechet@activ-france.fr](mailto:stephane.bechet@activ-france.fr) |
| Délégué à la protection des données | Structure | DPO consulting |
|  | Mail : | [dpo@chicreteil.fr](mailto:dpo@chicreteil.fr) |

**Sommaire**

[1 Page de signature du protocole 5](#_Toc57645901)

[2 Abréviations 6](#_Toc57645902)

[3 Résumé du projet 7](#_Toc57645903)

[4 Justification et contexte de la recherche 10](#_Toc57645904)

[4.1 Hypothèse de la recherche 10](#_Toc57645905)

[4.2 Description de la pathologie 11](#_Toc57645906)

[4.3 Description de la population concernée 12](#_Toc57645907)

[4.4 Bénéfice(s) 12](#_Toc57645908)

[4.5 Risque(s) et contrainte(s) ajouté(s) à la recherche 12](#_Toc57645909)

[5 Objectifs de la recherche 12](#_Toc57645910)

[5.1 Objectif principal 12](#_Toc57645911)

[5.2 Objectifs secondaires 12](#_Toc57645912)

[6 Sélection des personnes 13](#_Toc57645913)

[6.1 Critères d’inclusion 13](#_Toc57645914)

[6.2 Critères de non inclusion 13](#_Toc57645915)

[6.3 Objectif d’inclusion 13](#_Toc57645916)

[7 Méthodologie de la recherche 14](#_Toc57645917)

[7.1 Critères d’évaluation principaux et secondaires 14](#_Toc57645918)

[7.1.1 Critère d’évaluation principal 14](#_Toc57645919)

[7.1.2 Critère(s) d’évaluation secondaire(s) 14](#_Toc57645920)

[8 Déroulement de l’étude 14](#_Toc57645921)

[8.1 Sélection des patients 14](#_Toc57645922)

[8.2 Déroulement de la recherche 15](#_Toc57645923)

[8.2.1 Schéma du circuit patient 15](#_Toc57645924)

[15](#_Toc57645925)

[8.2.2 Détails des visites 15](#_Toc57645926)

[8.2.3 Calendrier des visites 16](#_Toc57645927)

[8.2.4 Détail des bilans et évaluations de l’étude 17](#_Toc57645928)

[8.3 Planning de la recherche : 19](#_Toc57645929)

[9 Evaluation de la sécurité 19](#_Toc57645930)

[9.1 Comité de surveillance 19](#_Toc57645931)

[10 Description des règles d’arrêt définitif ou temporaire 19](#_Toc57645932)

[10.1 Arrêt de la participation d’une personne à la recherche 19](#_Toc57645933)

[10.2 Arrêt d’une partie ou de la totalité de la recherche par le promoteur. 20](#_Toc57645934)

[11 Gestion des données 20](#_Toc57645935)

[11.1 Données recueillies 20](#_Toc57645936)

[11.2 Droits d’accès aux données 20](#_Toc57645937)

[11.3 Confidentialité 20](#_Toc57645938)

[11.4 Archivage 21](#_Toc57645939)

[11.5 Contrôle et Assurance qualité 21](#_Toc57645940)

[12 Aspects statistiques 21](#_Toc57645941)

[12.1 Evaluation du nombre de sujets à recruter 21](#_Toc57645942)

[12.2 Analyses 21](#_Toc57645943)

[13 Aspects éthiques et légaux 22](#_Toc57645944)

[13.1 Obligations légales 22](#_Toc57645945)

[13.2 Promoteur 22](#_Toc57645946)

[13.3 Soumission au CPP 22](#_Toc57645947)

[13.4 Modifications substantielles 22](#_Toc57645948)

[13.5 Données informatisées - CNIL 22](#_Toc57645949)

[13.6 Assurance 23](#_Toc57645950)

[14 Règles de publication 23](#_Toc57645951)

[14.1 Communications scientifiques 23](#_Toc57645952)

[14.2 Communication des résultats aux participants 23](#_Toc57645953)

[14.3 Cession des données 23](#_Toc57645954)

[15 Références bibliographiques 24](#_Toc57645955)

[16 ADDENDA 25](#_Toc57645956)

[16.1 Liste des centres participants 25](#_Toc57645957)

# Page de signature du protocole

| Acronyme de l’étude | SEINE (***SE****quella* ***I****n* ***NE****isseria*) |
| --- | --- |
| Titre de l’étude | Séquelles à long terme des méningites et des purpura fulminans à méningocoques de l’Enfant en Ile de France : une approche multidisciplinaire |
| Numéro ID-RCB | **2020-A00857-32** |

| Promoteur | | |
| --- | --- | --- |
| Centre Hospitalier Intercommunal de Créteil  40, avenue de Verdun  94010 Créteil cedex | Nom | Vauconsant Catherine  Directrice Générale |
|  | A Créteil, le |  |
|  | Signature : |  |

| Investigateur coordonnateur | | |
| --- | --- | --- |
| Centre Hospitalier Intercommunal de Créteil  40 avenue de Verdun,  94010 Créteil Cedex | Nom | Cohen Robert |
|  | A Créteil, le |  |
|  | Signature : |  |

# Abréviations

| ACTIV | Association clinique thérapeutique infantile du Val de Marne |
| --- | --- |
| ARC | Attaché de recherche Clinique |
| CHIC | Centre Hospitalier Intercommunal de Créteil |
| CRC | Centre de Recherche Clinique |
| IP | Investigateur principal |
| RIPH | Recherche interventionnelle sur la personne humaine |
| TEC | Technicien d’études cliniques |
| GPIP | Groupe de Pathologie Infectieuse Pédiatrique |

# Résumé du projet

| **Caractéristiques générales** | | |
| --- | --- | --- |
| Acronyme de l’étude | SEINE | |
| Numéro ID-RCB | **2020-A00857-32** | |
| Numéro ClinicalTrials | NCT04685850 | |
| Promoteur | Centre Hospitalier Intercommunal de Créteil | |
| Réglementaire | | |
| Type d’étude | Etude à risques et contraintes minimes impliquant la personne humaine (RIPH2) | |
| Nombre de centres | 56 services hospitaliers d’Ile de France | |
| Méthodologie | Etude multicentrique, prospective, non randomisée portant sur des patients ayant eu une infection invasive à méningocoques (IIM) de type méningite ou purpura fulminans à méningocoque diagnostiquée dans un service pédiatrique hospitalier en Ile de France entre 2010 et 2019. | |
| **Conception de la recherche** | | |
| Population concernée | Enfant de 1 à 15 ans ayant eu une méningite ou un purpura fulminans à méningocoque entre 2010 et 2019 | |
| Justification et contexte | La survenue d’IIM et principalement des méningites inquiète non seulement les professionnels de santé mais aussi la population générale où cette pathologie reste à juste titre une crainte majeure. Grâce aux réseaux de surveillance soutenus par Santé Publique France (CNR des méningocoques, réseau Epibac), on dispose en France de données d’incidence et de mortalité fiables. Il n’en va pas de même pour les séquelles. L’observatoire des méningites bactériennes de l’enfant mis en place en 2001 par le Groupe de Pathologie Infectieuse Pédiatrique (GPIP) permet du fait de sa méthodologie une approche à court terme des séquelles. En revanche l’évaluation des séquelles à plus long terme tant sur le plan clinique, que sur le développement intellectuel, sensoriel et des apprentissages nécessiterait un suivi des patients au minimum 1 an après la survenue de la pathologie.  L’observatoire des méningites bactériennes de l’enfant a permis d’identifier de 2001 à 2019, 7624 méningites et purpura fulminans tous germes confondus grâce à la participation de 233 services de pédiatrie. En Ile de France, 56 services de pédiatrie générale, de néonatalogie et de réanimation pédiatriques ont inclus 22% de cette cohorte. En considérant la période de 2010 à 2019, en Ile de France, 169 méningites et purpura fulminans à méningocoque ont été identifiés (15 à 20 cas par an), 9 patients sont décédés, 36 ont eu des séquelles à court terme, 21 des complications neurologiques, 8 des complications circulatoires et 7 ont présenté d’autres atteintes. Un sondage récent effectué auprès des services concernés en Ile de France a permis de constater que les patients sont revus en moyenne 1 an après l’épisode et qu’un certain nombre d’entre eux ont des séquelles (intellectuelles ou physiques) non identifiées initialement au moment de la sortie du patient de l’hôpital.  Ainsi, en sélectionnant uniquement la région Ile de France (représentative du reste de la France), les données de l’observatoire pourraient être utilisées pour la mise en place d’une étude prospective qui permettrait d’approcher plus finement les séquelles à long terme des méningites et purpura fulminans à méningocoque chez l’enfant avec notamment une évaluation, clinique, psychique, psychomotrice et orthophonique. De plus, une enquête auprès des parents devrait permettre d’apprécier leur vécu de la maladie, d’identifier la présence d’un stress post traumatique et de faire le point sur le parcours de soins mis en place (ou non) depuis la sortie de l’hôpital de leur enfant. Ainsi, la perception par l’entourage des conséquences de ces pathologies graves comme les amputations, leurs prises en charge médicale et logistique pourraient également être décrites. | |
| Retombées attendues | Peu d’études ont rapporté les séquelles à long terme des IIM telles que les méningites et les purpura fulminans chez l’enfant, au minimum 1 an après la sortie de l’hôpital et au maximum 10 ans après. En termes de santé publique, cette étude devrait mettre en évidence l’importance d’un parcours de soin « balisé » dans ces pathologies graves aux conséquences parfois tardives. | |
| Calcul du nombre de patients | Entre 2010 et 2019, 169 méningites et purpura fulminans à méningocoque ont été déclarés dans l’Observatoire national des Méningites Bactériennes de l’enfant. Après avoir exclus les patients décédés (n=9) et les patients perdus de vue (environ 20%, n=30) et en considérant un taux d’acceptation de 80%, 100 patients devraient être inclus et bénéficier du parcours de soin établi dans le cadre de l’étude. | |
| Objectif principal | Evaluer à long terme (entre 1 et 10 ans) les séquelles physiques, neurologiques ou sensorielles d’une méningite ou d’un purpura fulminans à méningocoque | |
| Critère d’évaluation principal | - Nombre d’enfants présentant au moins une séquelle neurologique, orthopédique ou sensorielle | |
| Objectifs secondaires | - Etablir un parcours de soin au-delà de 1 an de suivi - Evaluation du stress post traumatique parental - Evaluer l’impact sociétal de la maladie : scolarisation des enfants ; activité des parents | |
| Critères d’évaluation secondaires | - Nombre d’enfants ayant suivi le parcours de soins balisé avec établissement d’un score en fonction des consultations réalisées - Nombre d’enfants avec séquelles neurologiques graves : hémiplégie … - Nombre d’enfants avec complications orthopédiques (amputations…) - Nombre d’enfants avec des troubles sensoriels - Nombre d’enfants avec des séquelles cutanées - Nombre d’enfants ayant des difficultés d’apprentissage (sans séquelles neurologiques identifiées) - Nombre d’enfants ayant des difficultés cognitives ou psychologiques identifiés sur la WPPSI, WISC V ou VINELAND - Parcours de soins suivis par les enfants : Nombre de consultations et typologie du suivi (Consultations pédiatriques spécialisées ou non ; bilan/suivi ORL et orthophonique ; bilan/suivi ophtalmologique ; bilan neuro-développemental) - Evaluation du stress post traumatique des parents par le questionnaire IES-R | |
| Critères d’inclusion | - Nourrissons et enfants de 1 à 15 ans - Ayant eu une méningite ou un purpura fulminans à méningocoque inclus dans l’observatoire entre 2010 et 2019 - Les titulaires de l’autorité parentale ont lu et compris la lettre d’information et leur consentement exprès a été recueilli - Patient affilié à un régime de sécurité sociale (Sécurité Sociale ou Couverture Médicale Universelle) | |
| Critères de non inclusion | - Refus d’un des deux parents | |
| Déroulement de l’étude | | |
| Circuit patient |  | |
| Calendrier de l’étude | Date prévue du début de l’étude | Q4 2020 |
|  | Date prévue de fin des inclusions | Q2 2023 |
|  | Durée de la période d’inclusion | 2 ans |
|  | Durée de suivi d’un patient dans l’étude | 1 an |
|  |  | |
| Publications/ Communications | Publication dans un journal médical avec comité de lecture  Présentation (orale ou poster) de l’étude en congrès | |

# Justification et contexte de la recherche

Hypothèse de la recherche

La survenue d’infections invasives à méningocoques (IIM) et principalement des méningites inquiète non seulement les professionnels de santé mais aussi la population générale où cette pathologie reste à juste titre une crainte majeure. Grâce aux réseaux de surveillance soutenus par Santé Publique France (CNR des méningocoques, réseau Epibac), on dispose en France de données d’incidence et de mortalité fiables. Il n’en va pas de même pour les séquelles. L’observatoire des méningites bactériennes de l’enfant mis en place en 2001 par le Groupe de Pathologie Infectieuse Pédiatrique (GPIP) permet du fait de sa méthodologie une approche à court terme des séquelles. En revanche, l’évaluation des séquelles à plus long terme tant sur le plan clinique que sur le développement intellectuel, sensoriel et des apprentissages nécessiterait un suivi des patients au minimum 1 an après la survenue de la pathologie.

L’observatoire des méningites bactériennes de l’enfant a permis d’identifier de 2001 à 2019, 7624 méningites et purpura fulminans tous germes confondus grâce à la participation de 233 services de pédiatrie. En Ile de France, 56 services de pédiatrie générale, de néonatalogie et de réanimation pédiatriques ont inclus 22% de cette cohorte. En considérant la période de 2010 à 2019, en Ile de France, 169 méningites et purpura fulminans à méningocoque ont été identifiés (15 à 20 cas par an), 9 patients sont décédés, 36 ont eu des séquelles à court terme, 21 des complications neurologiques, 8 des complications circulatoires et 7 ont présenté d’autres atteintes. Un sondage récent effectué auprès des services concernés en Ile de France a permis de constater que les patients sont revus en moyenne 1 an après l’épisode et qu’un certain nombre d’entre eux présente des séquelles (intellectuelles ou physiques) non identifiées initialement au moment de la sortie de l’hôpital.

Ainsi, en sélectionnant uniquement la région Ile de France (considérée comme représentative du reste de la France), les données de l’observatoire pourraient être utilisées pour la mise en place d’une étude prospective qui permettrait d’approcher plus finement les séquelles à long terme des méningites et purpura fulminans à méningocoque chez l’enfant avec notamment une évaluation, clinique, psychique, psychomotrice et orthophonique. De plus, une enquête auprès des parents devrait permettre d’apprécier leur vécu de la maladie, d’identifier la présence d’un stress post traumatique et de faire le point sur le parcours de soins mis en place (ou non) depuis que leur enfant est sorti de l’hôpital. Ainsi, cette étude permettrait d’évaluer les conséquences de ces pathologies graves avec leurs prises en charge médicale et logistique mais également l’impact qu’elles ont sur les proches.

L’objectif principal de cette étude est d’évaluer à long terme les séquelles des IIM de type méningites et purpura fulminans chez l’enfant par une approche multidisciplinaire à la fois psychologique, intellectuelle, sensorielle et clinique. L’objectif secondaire est d’apprécier les conséquences sociétales, la perception de cette pathologie grave par l’entourage afin que le fardeau réel de la maladie puisse être mesuré justement en tenant compte de paramètres jusqu’à présent non pris en compte.

Description de la pathologie

Les IIM comprennent toutes les infections graves liées au méningocoque ou *Neisseria meningitidis* (Nm). L’infection invasive est définie par l’isolement du germe dans un espace normalement stérile (sang, liquide cérébrospinal...) et les deux principales sont les méningites et les septicémies (1). L’évolution peut se faire vers un choc septique dont le purpura fulminans, la forme la plus létale de la maladie, qui est caractérisé cliniquement par un purpura nécrotique et ecchymotique rapidement extensif associé à une insuffisance circulatoire (2).

Nm est une bactérie spécifique à l'homme et colonise la muqueuse des voies respiratoires supérieures de l'homme sans provoquer d’IIM chez environ 10 % de la population générale (3). Dans de rares cas, certaines souches de Nm, dites hyper invasives (4), peuvent traverser l’épithélium respiratoire, envahir la circulation sanguine et s’y multiplier causant une IIM.

Dans les pays industrialisés (Europe, Amérique et Océanie), l’incidence annuelle varie de 0,7 à 2 cas pour 100 000 habitants et elles sévissent essentiellement sous forme de cas sporadiques avec prédominance des souches de sérogroupes B et C. Ainsi, en France, l’incidence des IIM reste faible (proche du taux de 1/100 000 observé en moyenne en Europe) mais est plus élevée chez les nourrissons de moins de 1 an (8,9/100 000 en 2015) et les jeunes adultes de 18-20 ans (1,5/100 000 en 2015) (5). La majorité des cas surviennent de manière sporadique et sont majoritairement liés aux sérogroupes B qui représente 62% des cas en moyenne (5).

Il s’agit d’une pathologie sévère puisque le taux de mortalité est de 8 à 10%, ce qui représente 50 à 60 décès par an, (5) et peut atteindre des taux beaucoup plus élevé en cas de purpura fulminans (6). En termes de séquelles, les séquelles immédiates sont bien connues mais il existe peu d’étude s’intéressant aux séquelles à long terme et au développement cognitif des enfants. Les séquelles secondaires à une IIM en fin d’hospitalisation ont été rapportées dans 10 à 40 % des cas selon les études (7–10). Elles sont le plus souvent cutanées (nécrose cutanée avec perte de peau plus au moins étendue pouvant nécessité une greffe cutanée), orthopédiques (amputations), neurologiques (déficit neurologique focal, épilepsie, spasticité, surdité), cognitives (difficultés d’apprentissage scolaire), comportementales et psychologiques. Les séquelles les plus fréquentes chez les enfants ayant eu une IMM semblent être les nécroses cutanées (10 %) et les problèmes neurologiques de tout type (10 à 12,2 %) (11).

Toutefois, il existe peu d’études prospectives s’intéressant aux conséquences neuropsychologiques et développementales de ces enfants à distance et le taux de séquelles à distance est possiblement sous-estimée à l’heure actuelle. L’étude AMEND qui est actuellement en cours évalue les séquelles à distance chez les jeunes adultes (12) mais une étude prospective chez l’enfant est également nécessaire. En effet, cette dernière parait essentielle pour pouvoir évaluer de manière approfondie l’impact sur le développement que peuvent avoir ces IIM, pour avoir une estimation plus fiable du taux séquelles et surtout pour pouvoir mettre en place un suivi adapté de ces enfants.

Description de la population concernée

Cette étude concernera des enfants âgés de 0 à 15 ans ayant eu une méningite ou un purpura fulminans à méningocoque diagnostiquée dans un service pédiatrique hospitalier en Ile de France entre 2010 et 2019.

Bénéfice(s)

Peu d’études ont rapporté les séquelles à long terme des méningites et purpura fulminans à méningocoque chez l’enfant, au minimum 1 an après la sortie de l’hôpital et au maximum 10 ans après. En termes de santé publique, cette étude devrait mettre en évidence l’importance d’un parcours de soin « balisé » dans ces pathologies graves aux conséquences parfois tardives.

Pour les parents, cette étude leur permettra d’avoir accès à des spécialistes, sans engendrer de frais, leur permettant d’identifier si leur enfant est atteint de séquelles et permettant la mise en place du suivi correspondant, pour améliorer la prise en charge de l’enfant.

Risque(s) et contrainte(s) ajouté(s) à la recherche

L’étude ne comporte aucun risque. Les seules contraintes sont la nécessité de se rendre aux consultations des différents spécialistes.

# Objectifs de la recherche

Objectif principal

Évaluer à long terme (entre 1 et 10 ans) les séquelles physiques, neurologiques ou sensorielles d’une méningite ou d’un purpura fulminans à méningocoque.

Objectifs secondaires

- Établir un parcours de soins cadré
- Évaluer le stress post-traumatique parental à moyen/long terme
- Évaluer l’impact sociétal de la maladie : scolarisation des enfants ; activité des parents

# Sélection des personnes

Critères d’inclusion

- Nourrissons et enfants de 1 à 15 ans ;
- Ayant eu une méningite ou un purpura fulminans à méningocoque inclus dans l’observatoire entre 2010 et 2019 ;
- L’un des titulaires de l’autorité parentale a lu et compris la lettre d’information et dont le consentement exprès a été recueilli ;
- Patient affilié à un régime de sécurité sociale (Sécurité Sociale ou Couverture Médicale Universelle).

Critères de non inclusion

- Refus d’un des deux parents.

Objectif d’inclusion

Il est prévu d’inclure environ 100 patients dans l’étude.

# Méthodologie de la recherche

Il s’agit d’une étude interventionnelle à risques et contraintes minimes impliquant la personne humaine (RiPH2).

Cette étude est multicentrique, prospective et non randomisée.

Critères d’évaluation principaux et secondaires

### Critère d’évaluation principal

- Nombre d’enfant avec au moins une séquelle neurologique, cutanée, orthopédique ou sensorielle.

### Critère(s) d’évaluation secondaire(s)

- Nombre d’enfants ayant suivi le parcours de soins balisé avec établissement d’un score en fonction des consultations réalisées.
- Nombre d’enfants avec des séquelles neurologiques graves : hémiplégie …
- Nombre d’enfants avec complications orthopédiques (amputations…).
- Nombre d’enfants avec des troubles sensoriels.
- Nombre d’enfant avec des séquelles cutanées.
- Nombre d’enfants avec des difficultés d’apprentissage (sans séquelles neurologiques identifiées).
- Nombre d’enfants avec des difficultés cognitives ou psychologiques identifiés sur la WPPSI, WISC V ou VINELAND. Parcours de soins suivis par les enfants : Nombre de consultation et typologie du suivi ; Consultations pédiatriques spécialisées ou non ; bilan/suivi ORL et orthophonique ; bilan/suivi ophtalmologique ; bilan neuro-développemental.
- Evaluation du stress post traumatique des parents par le questionnaire IES-R.

# Déroulement de l’étude

Sélection des patients

L’étude sera proposée aux parents des enfants déclarés dans l’observatoire national des Méningites Bactériennes de l’enfant, non décédés et non perdus de vue. En cas d’acceptation de participation au protocole et après recueil du formulaire de consentement éclairé, les enfants seront inclus.

Déroulement de la recherche

### Schéma du circuit patient

**Identification du patient à inclure dans l’observatoire**

**Convocation par le pédiatre de l’enfant + parents**

Inclusion

**6**

**6**

**M**

**O**

**I**

**S**

**Consultation d’inclusion** (au moins 1 heure)

Examen clinique et recueil du parcours suivi de l’enfant

**Si Patient déjà suivi :**

Récupération des bilans ± proposition des tests de l’étude si certains manquent

**Si Patient non suivi** :

Organisation des 4 visites avec les spécialistes dans les 6 mois (max)

ORL Audiométrie

Ophtalmologiste

Psychologue

Tests selon âge

Orthophoniste

Tests selon âge

Remplissage Auto-questionnaire IES-R

par les parents

Récupération des résultats / bilans des différents spécialistes

**Consultation de fin d’étude**

Point parents enfant sur les bilans effectués

### Détails des visites

- **Visite d’inclusion**

Cette consultation d’inclusion avec le pédiatre investigateur durera au moins 1 heure et elle comportera :

- Interrogatoire sur les antécédents médicaux en lien avec la méningite (séquelles cutanées, notion d’hospitalisation depuis la méningite) et suivis mis en place.
- Examen clinique général, orthopédique, cutané.
- Examen neurologique.
- Les informations de suivi seront récupérées par le centre recruteur : bilans de consultation / hospitalisation ou CR d’imagerie (IRM cérébral si réalisé).
- Parcours scolaire de l’enfant, recherche de difficultés d’apprentissage.
- **Evaluations spécialisées, 4 au maximum**

Les évaluations spécialisées auront pour objectif d’évaluer les séquelles sensorielles éventuelles :

- Examen ORL et audiométrie.
- Examen visuel par un ophtalmologiste.
- Recherche de troubles du langage oral et écrit par une orthophoniste.
- Le développement intellectuel des enfants sera évalué par un WISC IV ou V effectué par une psychologue. En l’absence de réalisation possible du WISC (retard trop important), l’échelle de Vineland sera utilisée pour évaluer le développement de l’enfant.

Si l’enfant n’est pas suivi par un ou plusieurs spécialistes, une liste de professionnelle sera proposée aux parents de l’enfant pour une prise de RDV dans les 6 mois suivant la visite d’inclusion.

Si l’enfant est suivi, les résultats des évaluations antérieures seront colligés et, si besoin, complétées par le professionnel suivant l’enfant.

- **Visite de fin d’étude avec le pédiatre référent**

Cette consultation permettra au médecin hospitalier suivant l’enfant de faire un bilan avec les parents et un retour sur les bilans spécialisés effectués et la prise en charge possible.

*Evaluation du stress post-traumatique des parents* :

Le stress post-traumatique des parents sera évalué par le questionnaire Impact of Event Scale-Revisited (IES-R). Les parents pourront remplir ce questionnaire en ligne sur un site dédié.

### Calendrier des visites

| Acte/examen | Inclusion | période de 6 mois | Visite de fin d’étude |
| --- | --- | --- | --- |
| Signature du consentement éclairé | (*)✓ |  |  |
| Bilan pédiatrique/neurologique | ✓ |  | (*)✓ |
| Bilan psychologique |  | ✓ |  |
| Bilan orthophonique |  | ✓ |  |
| Consultation ORL (audiométrie) |  | ✓ |  |
| Consultation ophtalmologique (vision) |  | ✓ |  |
| Evaluation stress post-traumatique des parents |  |  | (*)✓ |

*(*) Ces actes/examen sont pratiqués dans le cadre de la recherche et non effectués lors de la pratique clinique habituelle*

### Détail des bilans et évaluations de l’étude

Si des bilans et/ou examens de l’enfant datent de plus de 1 an, ils seront refaits.

- **Bilan pédiatrique**

Interrogatoire détaillé, examen clinique général, cutané, orthopédique et neurologique. Parcours médical et scolaire, type de suivi mis en place.

- **Bilan orthophonique**

Des tests différents seront proposés à l’enfant en fonction de son âge

- Avant 4 ans : **EVALO 2-6,** batterie d’évaluation du développement du langage oral chez l’enfant de 2 ans 3 mois à 6 ans 3 mois.

Elle se décompose en 2 versions : version « petits » jusqu’à 4 ans 3 mois et version « grands » pour les plus âgés. Grâce à un éventail d’épreuves regroupées par domaines, elle permet d’explorer les différentes compétences séparément (compétences linguistiques, compétences pragmatiques, architecture fonctionnelle du langage) tant sur le versant expressif que sur celui de la compréhension.

- Après 4 ans : **Batterie informatisée EXALANG** de la plateforme HAPPYNEURON.

Elle est en fait constituée de 5 batteries, qui permettent un examen du langage oral, du langage écrit et des compétences transversales, pour les enfants de 3 à 20 ans.

Elle se décompose en tranches d’âge : 3-6 ans, 5-8 ans, 8-11 ans, 11-15 ans.

Les tests de chaque batterie sont randomisés, ce qui permet de sélectionner à la fois les épreuves que l’on souhaite faire passer (aucune obligation d’utiliser une batterie entière) et de choisir l’ordre de passation. Les épreuves sont normées.

Cet outil permet de coupler les données analysées à l'observation clinique. Les résultats sont sauvegardés sur un serveur sécurisé, agréé "données de santé ».

Durée du bilan : 1h30.

- **Bilan du développement/psychologique**

Des tests différents seront proposés à l’enfant en fonction de son âge avec une évaluation des troubles des apprentissages ainsi que des troubles cognitifs afin d’établir un score final.

- - **WPPSI IV**(Entre 2.5 ans et 6 ans) :

Evaluation du Qi dans plusieurs dimensions : compréhension verbale, visuo-spatiale, raisonnement fluide, mémoire de travail, vitesse de traitement.

- - **WISC V**(A partir de 6 ans) :

Evaluation du Qi comprenant un indice de compréhension verbale, un indice visuo-spatial, un indice de raisonnement fluide, un indice de mémoire de travail et un indice de vitesse de traitement.

Un score total≤70 est associé à un retard mental ; un score compris entre 70 et 130 à une intelligence faible à supérieure ; supérieur à 130 un haut potentiel intellectuel.

- - **Echelles de Vineland** (évaluation des comportements) se fait à tous les âges, et sera proposé pour les enfants pour lesquels le WISC ou WPPSI ne seront pas réalisables. Le développement sera apprécié sur une échelle de développement basée sur l’interrogatoire des parents.

Age de développement et écart type par rapport à l’âge chronologique.

Durée du bilan : au moins 1h30.

- **Bilan ophtalmologique**

L’examen permettra de déterminer la présence de troubles de la réfraction, de strabisme,

d’amblyopie et/ou de troubles ophtalmologique moteurs.

Un bilan orthoptique éventuel pourra être nécessaire si prescrit par l’ophtalmologiste.

Durée du bilan : 20 min.

- **Bilan ORL**

Réalisation d’un audiogramme (à refaire si l’audiogramme date de plus de 1 an).

Durée du bilan : 10 min.

- **Evaluation post traumatique des parents**

L’IES-R est un auto-questionnaire comprenant 22 questions et validé en Français. Il permet d’évaluer le ressenti parental face à la maladie de leur enfant et d’identifier la présence d’un stress post traumatique (score total ≥33).

Cette enquête sera proposée en ligne.

Planning de la recherche :

| Date de début des inclusions : | Q4 2020 |
| --- | --- |
| Durée des inclusions : | 24 mois |
| Date de fin de suivi des patients : | Q4 2023 |
| Date de publication des résultats : | Q2 2024 |
| Durée de participation d’un sujet : | 12 mois |

# Evaluation de la sécurité

Selon la législation française, la vigilance des études à risques et contraintes minimes correspond à ceux réalisés dans le cadre des soins habituels. Les événements indésirables seront signalés à la pharmacovigilance, à la matériovigilance ou à la biovigilance de l'organisme correspondant. A noter que dans cette étude aucun médicament n’est testé. Une surveillance des effets indésirables attendus sera spécifiquement effectuée pour l'étude.

Comité de surveillance

Compte-tenu de la nature de l’étude (étude interventionnelle à risques et contraintes minimes), il n’est pas prévu de comité de surveillance.

# Description des règles d’arrêt définitif ou temporaire

Arrêt de la participation d’une personne à la recherche

Les patients inclus dans l’étude peuvent demander à sortir de l’étude à n’importe quel moment et quelle qu’en soit la raison.

L’investigateur peut interrompre temporairement ou définitivement la participation d’un patient à l’étude pour toute raison qui servirait au mieux les intérêts du patient.

La sortie d’étude d’un patient ne changera en rien la prise en charge habituelle.

En cas de patient perdu de vue, l’investigateur met tout en œuvre pour reprendre contact avec le patient. En cas de sortie prématurée d’étude, l’investigateur doit en documenter les raisons de façon aussi complète que possible dans le dossier médical.

Les données recueillies pour les patients perdus de vue ou sortis prématurément de l’étude seront exploitées au moment des analyses.

En cas de retrait du consentement et conformément à la réglementation, les données seront utilisées jusqu’à la date de retrait du consentement.

Arrêt d’une partie ou de la totalité de la recherche par le promoteur.

Des événements imprévus, au vu desquels les objectifs de l’étude ne seront vraisemblablement pas atteints, peuvent amener le promoteur à interrompre prématurément l’étude.

Le promoteur se réserve le droit d’interrompre l’étude, à tout moment, s’il s’avère que les objectifs d’inclusion ne sont pas atteints.

En cas d’arrêt prématuré de l’étude, l’information sera transmise par le promoteur dans un délai de 15 jours au CPP.

# Gestion des données

Données recueillies

Pour chaque patient inclus, les données suivantes seront colligées :

- Antécédents médicaux
- Examen clinique
- Scolarité
- Résultats de l’audiométrie, examen visuel
- Résultats évaluation orthophonique, psychologique
- Parcours scolaire, prises en charge sociale
- Résultats questionnaire de stress post traumatique parental

L’ensemble des données recueillies seront colligées dans un premier temps sur un questionnaire papier puis les données seront saisies dans un cahier d’observation électronique.

Droits d’accès aux données

Les investigateurs mettront à disposition les documents et données individuelles strictement nécessaires au suivi, au contrôle de qualité et à l’audit de la recherche, à la disposition des personnes ayant accès à ces documents conformément aux dispositions législatives et réglementaires en vigueur (Articles L.1121-3 et R.5121-13 du code de la santé publique).

Confidentialité

Conformément aux dispositions législatives en vigueur (articles L.1121-3 et R.5121-13 du code de la santé publique), les personnes ayant un accès direct aux données source prendront toutes les précautions nécessaires en vue d'assurer la confidentialité des informations relatives à la recherche, aux personnes qui s'y prêtent et notamment en ce qui concerne leur identité ainsi qu’aux résultats obtenus. Ces personnes, au même titre que les investigateurs eux-mêmes, sont soumises au secret professionnel.

Chaque patient se verra attribuer un code d’identification composé d’un numéro de centre et d’un numéro de patient (3 chiffres).

Le promoteur s’assurera que chaque personne qui se prête à la recherche a donné son accord par écrit pour l’accès aux données individuelles la concernant et strictement nécessaire au contrôle de qualité de la recherche.

Archivage

Les documents suivants relatifs à la recherche sont archivés conformément aux Bonnes Pratiques Cliniques et à la réglementation en vigueur pour une durée de 15 ans suivant la fin de la recherche par le promoteur.

Contrôle et Assurance qualité

Préalablement à l’analyse des données, une recherche de valeurs aberrantes, incohérences et données manquantes sera effectuées. L’investigateur sera recontacté pour correction éventuelle. Il n’y aura pas de monitoring sur site des données.

Un audit peut être réalisé à tout moment par des personnes mandatées par le promoteur et indépendantes des responsables de la recherche. Il a pour objectif de s’assurer de la qualité de la recherche, de la validité des résultats et du respect de la loi et de la réglementation en vigueur.

Les investigateurs acceptent de se conformer aux exigences du promoteur et à l’autorité compétente en ce qui concerne un audit ou une inspection de la recherche.

# Aspects statistiques

Evaluation du nombre de sujets à recruter

Entre 2010 et 2019, 169 méningites et purpura fulminans à méningocoque ont été déclarés dans l’Observatoire national des Méningites Bactériennes de l’enfant. Les données cliniques initiales sont déjà connues pour ces patients. Après avoir exclus les patients décédés (n=9) et les patients perdus de vue (environ 20%, n=30) et en considérant un taux d’acceptation de 80%, 100 patients devraient être inclus et bénéficier du parcours de soins établi dans le cadre de l’étude.

Analyses

La proportion avec intervalle de confiance d’enfants avec séquelles sera décrite, pour l’ensemble de la cohorte recrutée, au sein de chaque classe d’âge, et en fonction du type de séquelles : neurologique, orthopédique, cutanée, sensorielle, développementale. Les données qualitatives seront décrites par leur nombre et pourcentage. Les données quantitatives par leur moyenne et écart type. Des courbes de survie type Kaplan Meier seront tracées pour déterminer la présence de séquelles à différents temps post méningite. Des tests de comparaisons pourront être effectués pour déterminer la présence de séquelles selon la gravité ou l’atteinte initiale.

# Aspects éthiques et légaux

Obligations légales

Le promoteur et les personnes qui dirigent et surveillent la recherche s’engagent à ce que cette recherche soit réalisée en conformité avec la loi n°2012-300 du 5 mars 2012 relative à la politique de santé publique et les dispositions réglementaires en vigueur. (Articles L1121-1, 2° alinéa et R1121-3 du Code de la santé publique).

La recherche est conduite conformément au présent protocole.

Promoteur

Le Centre hospitalier intercommunal de Créteil (CHIC) est le promoteur de cette recherche.

Soumission au CPP

Cette étude clinique a reçu l’avis favorable du CPP Sud Méditerranée 1 le 22/12/2020.

Modifications substantielles

Les demandes de modifications substantielles seront adressées par le promoteur pour avis auprès du comité de protection des personnes concerné.

Le protocole modifié devra faire l’objet d’une version actualisée datée.

Les formulaires d’information et de recueil de consentement du patient devront faire l’objet de modification si nécessaire.

Données informatisées - CNIL

Le traitement des données sera réalisé dans les conditions de confidentialité définies par la délibération no 2018-153 du 3 mai 2018 portant modification de la méthodologie de référence pour les traitements de données personnelles opérés dans le cadre des recherches impliquant la personne humaine (MR-001).

Assurance

Le promoteur, le CHI Créteil, déclare avoir souscrit une police d’assurance à la SHAM, police n°102.760, garantissant, selon les clauses prévues au contrat et dans la limite des sommes fixées, les conséquences pécuniaires de sa responsabilité civile telle qu’elle résulte de l’application de l’article L 1121-10 du code de la Santé Publique.

# Règles de publication

Communications scientifiques

Toute communication écrite ou orale des résultats de la recherche doit recevoir l’accord préalable du promoteur et de l’investigateur coordonnateur.

Les résultats principaux de cette étude seront publiés dans une revue internationale en anglais à comité de lecture. Sur les publications princeps et associées seront mentionnées les membres du comité scientifique et les investigateurs.

Le soutien financier de SANOFI pour la réalisation de cet essai sera mentionné.

Communication des résultats aux participants

Conformément à la loi n°2002-303 du 4 mars 2002, les participants sont informés, à leur demande, des résultats globaux de la recherche.

Cession des données

Le CHI de Créteil est propriétaire des données.

Les conditions de cession de tout ou partie de la base de données de la recherche sont décidées par le promoteur de la recherche et font l’objet d’un contrat écrit.

# Références bibliographiques

1. Nadel S, Ninis N. Invasive Meningococcal Disease in the Vaccine Era. Front Pediatr. 2018;6:321.

2. Chalmers E, Cooper P, Forman K, Grimley C, Khair K, Minford A, et al. Purpura fulminans: recognition, diagnosis and management. Arch Dis Child. nov 2011;96(11):1066‑71.

3. Yazdankhah SP, Caugant DA. Neisseria meningitidis: an overview of the carriage state. J Med Microbiol. sept 2004;53(Pt 9):821‑32.

4. Zarantonelli ML, Lancellotti M, Deghmane AE, Giorgini D, Hong E, Ruckly C, et al. Hyperinvasive genotypes of Neisseria meningitidis in France. Clin Microbiol Infect Off Publ Eur Soc Clin Microbiol Infect Dis. mai 2008;14(5):467‑72.

5. Parent du Chatelet I, Deghmane AE, Antona D, Hong E, Fonteneau L, Taha MK, et al. Characteristics and changes in invasive meningococcal disease epidemiology in France, 2006-2015. J Infect. juin 2017;74(6):564‑74.

6. Campsall PA, Laupland KB, Niven DJ. Severe meningococcal infection: a review of epidemiology, diagnosis, and management. Crit Care Clin. juill 2013;29(3):393‑409.

7. Pace D, Pollard AJ. Meningococcal disease: clinical presentation and sequelae. Vaccine. 30 mai 2012;30 Suppl 2:B3-9.

8. Ó Maoldomhnaigh C, Drew RJ, Gavin P, Cafferkey M, Butler KM. Invasive meningococcal disease in children in Ireland, 2001-2011. Arch Dis Child. déc 2016;101(12):1125‑9.

9. Huang L, Heuer OD, Janßen S, Häckl D, Schmedt N. Clinical and economic burden of invasive meningococcal disease: Evidence from a large German claims database. PloS One. 2020;15(1):e0228020.

10. Stein-Zamir C, Shoob H, Sokolov I, Kunbar A, Abramson N, Zimmerman D. The clinical features and long-term sequelae of invasive meningococcal disease in children. Pediatr Infect Dis J. juill 2014;33(7):777‑9.

11. Wang B, Clarke M, Thomas N, Howell S, Afzali HHA, Marshall H. The clinical burden and predictors of sequelae following invasive meningococcal disease in Australian children. Pediatr Infect Dis J. mars 2014;33(3):316‑8.

12. Marshall H, McMillan M, Wang B, Booy R, Afzali H, Buttery J, et al. AMEND study protocol: a case-control study to assess the long-term impact of invasive meningococcal disease in Australian adolescents and young adults. BMJ Open. 29 2019;9(12):e032583.

# ADDENDA

Liste des centres participants

| N° | Nom du centre | Service | Adresse du centre | Nom de l’investigateur principal | Prénom de l’investigateur principal |
| --- | --- | --- | --- | --- | --- |
| 1 | Hôpital Robert Debré | Pédiatrie générale | 48 Bd Sérurier 75019 Paris | FAYE | Albert |
| 2 | Groupe Hospitalier Nord Essonne | Pédiatrie | 159 rue du président F. Mitterrand 91345 Longjumeau | GASCHIGNARD | Jean |
| 3 | CHU paris Sud site Antoine Béclère | Pédiatrie Générale | 157 rue de la porte de trivaux92140 Clamart | MILCENT | Karen |
| 4 | CH Argenteuil | Pédiatrie Générale | 69 Rue du Lieutenant-Colonel Prudhon, 95107 Argenteuil | BENSAID | Philippe |
| 5 | CH F Quesnay | Pédiatrie | 2 boulevard Sully 78200 Mantes la Jolie | PELLEGRINO | Béatrice |
| 6 | GHEF site de Meaux | Pédiatrie | 6-8 rue Saint Fiacre 77100 Meaux | VIGNAUD | Olivier |
| 7 | Hôpital Necker Enfants Malades | Maladies Métaboliques Pédiatriques | 149 rue de Sèvres  75015 Paris | PICHARD | Samia |
| 8 | CHI de Créteil | Pédiatrie | 40 avenue de Verdun 94010 Créteil | MADHI | Fouad |
