# Supplementary material for: Long-term impact of invasive meningococcal disease in children: SEINE study protocol
Source: PLoS One. 2022 May 26;17(5):e0268536. doi: 10.1371/journal.pone.0268536 (PMC9135194; doi:10.1371/journal.pone.0268536)
Supplement: S4 File — (DOCX) [file pone.0268536.s004.docx]

Summary

[1 Patient Identification 2](#_Toc100156419)

[1.1 Identification 2](#_Toc100156420)

[2 Eligibility criteria 2](#_Toc100156421)

[2.1 Inclusion criteria 2](#_Toc100156422)

[2.2 Non-inclusion criteria 2](#_Toc100156423)

[3 Inclusion visit 3](#_Toc100156424)

[3.1 Methods of medical follow-up 3](#_Toc100156425)

[3.2 Clinical examination 3](#_Toc100156426)

[3.3 Educational background 5](#_Toc100156427)

[3.4 Everyday life 6](#_Toc100156428)

[4 Assessments of supplementary examinations 7](#_Toc100156429)

[4.1 Psychological and neurological assessment 7](#_Toc100156430)

[4.2 Speech therapy assessment 9](#_Toc100156431)

[4.3 ENT assessment / Audiometry 11](#_Toc100156432)

[4.4 Ophthalmological assessment 12](#_Toc100156433)

[4.5 Hospitalization 15](#_Toc100156434)

[5 Final visit: presentation of the assessments results 15](#_Toc100156435)

[5.1 Assessment of Post-Traumatic Stress of Parents (IES-R) 15](#_Toc100156436)

[6 End of study 15](#_Toc100156437)

# Patient Identification

| Identification | |
| --- | --- |
| 1. Patient number | \|__\| __\| __\| |
| 1. Last name of the patient (initial) | \|__\| |
| 1. First name of the patient (initial) | \|__\| |
| 1. Date of birth | \|__\|__\| / \|__\|__\| / \|__\|__\|__\|__\| |
| 1. Investigator's name |  |

# Eligibility criteria

| Inclusion criteria | **Yes** | **No** |
| --- | --- | --- |
| 1. Infant and child 0 to 15 years of age at the time of severe meningococcal infection |  |  |
| 1. Infant or child who had meningitis or meningococcal purpura fulminans included in the observatory between 2010 and 2019 |  |  |
| 1. The consent of the holders of authority has been obtained |  |  |
| 1. Patient affiliated to a social security scheme (Social Security or Universal Medical Coverage) |  |  |

| Non-inclusion criteria | **Yes** | **No** |
| --- | --- | --- |
| 1. Refusal of one of the two parents to participate in the study |  |  |

# Inclusion visit

| 1. Date of diagnosis of invasive meningococcal infection | \|__\|__\| / \|__\|__\| / \|__\|__\|__\|__\| |
| --- | --- |
| Methods of medical follow-up | |
| 1. Child followed since meningitis | Yes  No |
| 1. Frequency of consultations | \|__\|__\| consultations / year |
| 1. Pediatric follow-up | Yes  No |
| 1. Orthopedic follow-up | Yes  No |
| 1. Pediatric neuropediatric follow-up | Yes  No |
| 1. Psychological follow-up | Yes  No |
| 1. Psychiatric follow-up | Yes  No |
| 1. Psychomotor follow-up | Yes  No |
| 1. Speech-language monitoring | Yes  No |
| 1. ENT follow-up | Yes  No |
| 1. Ophthalmological follow-up | Yes  No |
| 1. Other follow-up | Yes  No |
| - 1. If so, which one |  |
| Clinical examination | |
| 1. Date of examination | \|__\|__\| / \|__\|__\| / \|__\|__\|__\|__\| |
| 1. Weight | \|__\|__\| kg ± \|__\|__\| DS |
| 1. Waist | \|__\|__\|__\| cm ± \|__\|__\| DS |
| 1. Cranial perimeter | \|__\|__\| cm ± \|__\|__\| DS |
| 1. Steady growth curve | Yes  No |
| - 1. Clarification if necessary |  |
| 1. ***Skin sequelae*** | Yes  No |
| - 1. If yes, which type |  |
| - 1. Localization |  |
| - 1. More than one site affected | Yes  No |
| - 1. History of skin grafting | Yes  No |
| 1. ***Orthopaedic sequelae*** | Yes  No |
| - 1. If so, amputation | Yes  No |
| - 1. Multiple member segments | Yes  No |
| - 1. Localization |  |
| - 1. History of orthopaedic surgery (excluding amputation) | Yes  No |
| - 1. Tendon retraction | Yes  No |
| - 1. Limb asymmetry | Yes  No |
| - - 1. If yes, describe: |  |
| 1. ***Neurological examinations*** | Yes  No |
| Evolution of the cranial perimeter curve | Normocephaly  Microcephaly  Macrocephaly |
| - 1. Chronic headaches | Yes  No |
| - 1. Epilepsy | Yes  No |
| - - 1. If so, type of epilepsy |  |
| - - 1. Start date | \|__\|__\| / \|__\|__\| / \|__\|__\|__\|__\| |
| - - 1. Current treatment |  |
| - - 1. Number of seizures in the last 6 months | \|__\|__\|__\| |
| - 1. Interaction with the environment | Yes  No |
| - - 1. If not, describe: |  |
| - 1. Axial tone | Normal  Hypotonia  Hypertonia |
| - 1. Normal object handling (can grab an object, move from one hand to the other, etc.) | Yes  No |
| - - 1. If not, describe: |  |
| - 1. Verbal communication | Yes  No |
| Swallowing disorders | Yes  No |
| - 1. Balance disorders | Yes  No |
| - - 1. If yes, describe: |  |
| - 1. Gait disorder | Yes  No |
| - - 1. If yes, describe: |  |
| - 1. Focal motor deficit | Yes  No |
| - - 1. If yes, describe: |  |
| - 1. Focal sensory deficit | Yes  No |
| - - 1. If yes, describe: |  |
| - 1. Abnormality Osteotendinous reflexes | Yes  No |
| - - 1. If yes, describe: |  |
| - 1. Signs of retraction (clonus, Achilles’ stiffness) | Yes  No |
| - - 1. If yes, describe: |  |
| - 1. Muscle tone | Normal  Abnormal |
| - - 1. If abnormal, right upper limb | Normal  Hypotonia  Hypertonia |
| - - 1. If abnormal, upper left limb | Normal  Hypotonia  Hypertonia |
| - - 1. If abnormal, right lower limb | Normal  Hypotonia  Hypertonia |
| - - 1. If abnormal, lower left limb | Normal  Hypotonia  Hypertonia |
| - 1. Gose-Ped Quotation | 7 – vegetative state  6 – severe disability: lower level  5 – severe disability: higher level  4 – moderate disability: lower level  3 – moderate disability: higher level  2 – good recovery: lower level  1 – good recovery: higher level |
| - 1. Specific motor equipment | Yes  No |
| - - 1. If so, which one | Orthotic  Prosthesis  Corset  Armchair  Other |
| - - 1. If necessary, specify the type of medical device |  |
| 1. ***Sensitive examinations and learning*** | Yes  No |
| - 1. Deafness | Yes  No |
| - - 1. If yes, hearing aid | Yes  No |
| - 1. Known language disorder | Yes  No |
| - 1. Known visual disorder | Yes  No |
| - - 1. If so, strabismus | Yes  No |
| - - 1. Other visual disturbance |  |
| - 1. Attention disorder | Yes  No |
| - 1. Behavioural disorder | Yes  No |
| - - 1. If so, which |  |
| - 1. Psychiatric disorder | Yes  No |
| - - 1. If so, autism spectrum disorder | Yes  No |
| - 1. Learning disability | Yes  No |
| - 1. Other psychiatric disorder, specify |  |
| - 1. Graphic disorder | Yes  No |
| - 1. Other disorder | Yes  No |
| - - 1. If so, which one |  |
| - 1. MDPH support (Departmental center for the disabled person) | Yes  No |
| - - 1. If yes, level of disability | <50%  50-79%  ≥80% |
| Educational background | |
| 1. Current class | Nursery or equivalent, nanny  Small kindergarten section  Middle section of kindergarten  Large kindergarten section  CP  EC1  THIRD GRADE  FOURTH GRADE  FIFTH GRADE  6th  5th  4th  3rd  2nd  1st  Terminal  Higher education  Not in school (child > 3 years old) |
| 1. Doubling | Yes  No |
| 1. Adapted schooling | Yes  No |
| - 1. ULIS (Localized unit for school inclusion) | Yes  No |
| - 1. IME (medical-educational institute) | Yes  No |
| - 1. SEGPA (adapted section of general and vocational education) | Yes  No |
| - 1. Distance learning (CNED: national center for distance learning) | Yes  No |
| - 1. Presence of an AVS (school life assistants), specialized educator | Yes  No |
| 1. School break (> 3 months) | Yes  No |
| Everyday life | |
| 1. Sleep quality with the Owl test (score /27) | \|__\|__\| |

# Assessments of supplementary examinations

|  | |
| --- | --- |
| Psychological and neurological assessment | |
| 1. Control brain imaging from meningitis | Yes  No |
| - 1. If so, what type of image | IRMc  TDMc  Other |
| - 1. If yes, date of last imaging | \|__\|__\| / \|__\|__\| / \|__\|__\|__\|__\| |
| - 1. If anomaly, which |  |
| 1. **WPPSI score (patient between 2 and 6 years old)**   ***(file to download in the eCRF)*** | Rolled  Not applicable  If completed, score: \|__\|__\|__\| |
| - 1. Date of the test | \|__\|__\| / \|__\|__\| / \|__\|__\|__\|__\| |
| 1. Attention and executive functions |  |
| 1. Language |  |
| 1. Memory and learning |  |
| 1. Sensorimotor functions |  |
| 1. Social perception |  |
| 1. Visuo-spatial treatments |  |
| 1. WPPSI score (patient between 2 and 6 years old) |  |
| 1. Conclusion of the cognitive test performed | ..................................................................................................................................................................................................................................................................................................................... |
| 1. **WISC V score (patient from 6 years old)**   ***(file to download in the eCRF*** | Rolled Not applicable  If completed, score: \|__\|__\|__\| |
| - 1. Date of the test | \|__\|__\| / \|__\|__\| / \|__\|__\|__\|__\| |
| 1. Verbal Comprehension Index |  |
| 1. Visuo-Spatial Index |  |
| 1. Fluid Reasoning Index |  |
| 1. Working Memory Index |  |
| 1. Processing Speed Index |  |
| 1. WISC V score (patient from 6 years old) |  |
| 1. Conclusion of the cognitive test performed | ........................................................................................................................................................................ ................................................................ |
| 1. **Vineland Scale** | Rolled  Not applicable  If completed, \|__\|__\| years ± \|__\|__\| DS |
| - 1. Date of the test | \|__\|__\| / \|__\|__\| / \|__\|__\|__\|__\| |
| 1. Conclusion of the cognitive test performed | .............................................................................................................................................................................................................................................................................................................................................................................. |

|  | |
| --- | --- |
| Speech therapy assessment | |
| Date of award | \|__\|__\| / \|__\|__\| / \|__\|__\|__\|__\| |
| 1. ***Oral language: EVALO (patient < 4 years old)*** | Normal  Abnormal  Not applicable |
| 43.1. Phonetic Test | Yes  No |
| 43.2. Phonology | Yes  No |
| 43.3. Glossary | Yes  No |
| 43.4. Morphosyntax- Expression | Yes  No |
| 43.5. Morphosyntax-Understanding | Yes  No |
| 43.6. Attention sounds | Yes  No |
| 43.7. Brief | Yes  No |
| 43.8. Auditivo-verbal gnosies | Yes  No |
| 43.9. Oral and lingual praxies | Yes  No |
| 43.10. Joint disorders | Yes  No |
| 43.11. Speech/language delay | Yes  No |
| 43.12. Severe disorder | Yes  No |
| 1. ***Feedback*** | ........................................................................................................................................................................................................................................................................................................................ |
| 1. ***Oral language: EXALANG (patient from 4 years old)*** | Normal  Abnormal  Not applicable |
| 45.1. Phonetic test | Yes  No |
| 45.2. Phonology | Yes  No |
| - 1. Lexicon | Yes  No |
| - 1. Difficulties Expression | Yes  No |
| - 1. Difficulties Understanding | Yes  No |
| - 1. Visual attention | Yes  No |
| - 1. Auditory attention | Yes  No |
| - 1. Memory | Yes  No |
| - 1. Oral and lingual praxia | Yes  No |
| - 1. Joint disorders | Yes  No |
| - 1. Speech/Language Delay | Yes  No |
| - 1. Severe disorder | Yes  No |
| - 1. Feedback | .................................................................................................................................................................................................................................................................................................................. |
| 1. ***Written language: EXALANG (patient from 5 years old)*** | |
| - 1. Not applicable |  |
| - 1. Difficulties in graphic design | Yes  No |
| - 1. Reading difficulties | Yes  No |
| 45.2.1. Dyslexia | Yes  No |
| - 1. Spelling difficulties | Yes  No |
| 45.3.1. Dysorthography | Yes  No |
| - 1. Logico-mathematical difficulties | Yes  No |
| - 1. Feedback | ..................................................................................................................................................................................................................................................................................................................... |

|  | | |
| --- | --- | --- |
| ENT assessment / Audiometry | | |
| 1. Audiometry | Yes  No | |
| - 1. If yes, date | \|__\|__\| / \|__\|__\| / \|__\|__\|__\|__\| | |
| - 1. Tonal abnormality   2. CO threshold in tonal   3. CA threshold in tonal | **Right ear:**  Yes  No  ......... ...... . Db  ............... .. Db | **Left Ear:**  Yes  No  .................dB  .................dB |
| - 1. Vocal anomaly   2. Intelligibility threshold | **Right ear:**  Yes  No  .................. Db | **Left Ear:**  Yes  No  .................. Db |
| 1. ENT Exam 2. Normal otoscopy | Yes  No  Yes  No | |
| 1. If abnormal otoscopy | **RIGHT EAR**  Retraction pocket  Otitis media  Serous otitis  EAC Anomaly  Other, prespecify ............  ................................................................................................................................................ | **LEFT Ear**  Retraction pocket  Serous otitis  Otitis media  EAC Anomaly  Other, specify:.........  ................................................................................................................................................ |
| - 1. Conclusion of the ENT exam | ............................................................................................................................................................................................................................................................................................................................................................................................. | |

|  | | | |
| --- | --- | --- | --- |
| Ophthalmological assessment | | | |
| 1. Ophthalmological assessment | Yes  No | | |
| - 1. If yes, date | \|__\|__\| / \|__\|__\| / \|__\|__\|__\|__\| | | |
| - 1. Ptosis | **OD:**  Yes  No  **OG:**  Yes  No | | |
| - 1. Photophobia | Yes  No | | |
| - 1. Direct and consensual photomotor reflex | Normal  Abnormal | | |
| - - 1. If abnormal, | DPAR  OTHER | | |
| - - - 1. If Other, specify: |  | | |
| - 1. Strabismus | Yes No  If YES:  **WITHOUT** OPTICAL CORRECTION:  **EXOPHORIA**  Yes  No  **EXOTROPY**  Yes  No  **ESOPHORIE**  Yes  No  **ESOTROPY**  Yes  No  **WITH** OPTICAL CORRECTION:  **EXOPHORIA**  Yes  No  **EXOTROPY**  Yes  No  **ESOPHORIE**  Yes  No  **ESOTROPY**  Yes  No | | |
| - 1. Nystagmus | Yes  No  If, yes:  Latent  Manifest | | |
| - 1. Eye motility | Normal  Limited, more details:................................. | | |
| - 1. Distance visual acuity | Yes  No | | |
| - - 1. **If yes,** Distance Visual Acuity (/10) | Method to be specified:  MONOYER  SELLEN  EDTRS  TUMBLING E  TUMBLING C  LANDOLT  LEA SYMBOLS  \|__\|__\|/ 10 **No** correction  \|__\|__\| / 10 **With** correction | | |
| - - 1. If yes, Near visual Acuity | PARINAUD scale  **OD**: \|__\|__\|__\|__\|  **OG**: \|__\|__\|__\|__\| | | |
| - - 1. **If not (impossible)** | **OD**  Smooth continuation:  yes  no  Occlusion resistance:  yes  no  Reflexes to the threat:  yes  No | **OG**  Smooth continuation:  yes  no  Occlusion resistance:  yes  no  Reflexes to the threat:  yes  No | |
| - 1. Test 15 HUE | Realized,  Not done  Specify:.......................................................................................................  If realized:  Saturated  Desaturated  Normal  Abnormal: If abnormal: Blue-yellow axis  Red green axis  If not realized, specify: ..................................................................................................... . | | |
| - 1. Stereoscopic vision Lang I | Yes  No | | |
| - - 1. If so, shorten the result | ....................................... . (SECOND ARC SOE) | | |
| - 1. Amblyopia | Yes No | | |
| - - 1. If so, Amblyopia | **OD:**  Moderated  Mild  Severe | | **OG:**  Moderated  Mild  Severe |
| - 1. Refractive disorder | Yes  No | | |
| - 1. Refraction with Cycloplegia   *(If possible)* | \|  \| **Sphere** \| **Cylinder** \| **Axis** \| \| --- \| --- \| --- \| --- \| \| **OD** \|  \|  \|  \| \| **OG** \|  \|  \|  \| | | |
| - 1. Eye tone   *(If possible)* | Yes  No | | |
| - - 1. If yes, specify | Intraocular tone OD (mmHg) \|__\|__\|  Og intraocular tone (mmHg) \|__\|__\| | | |
| - 1. Optic neuropathy | Papillary edema  Yes  No  Optical atrophy  Yes  No  **OTHER**  Yes  No | | |
| - - 1. If Other optic neuropathy, specify |  | | |
| - 1. Retinopathy | Yes  No | | |
| - - 1. If yes, specify the result of the retinophotograph if abnormality of the fundus of the eye | .......................................... . | | |
| - 1. **Optical coherence tomography** *(If possible)* | Yes  No  If yes:  MACULA : Normal ABNORMAL (specify).......  PAPILLA : Normal ABNORMAL (specify)....... | | |
| - 1. Other visual abnormality | Yes  No | | |
| - - 1. If other visual anomaly, detail | ............................................. . | | |

|  | |
| --- | --- |
| Hospitalization | |
| 1. Unscheduled re-hospitalization(s) since meningitis | Yes  No |
| - 1. If so, how much | \|__\|__\| |
| - 1. For what reason(s) |  |

# Final visit: presentation of the assessments results

| 1. Date of the visit | \|__\|__\| / \|__\|__\| / \|__\|__\|__\|__\| |
| --- | --- |
| Assessment of Post-Traumatic Stress of Parents (IES-R) | |
| 1. IES-R score *(done online)* | \|__\|__\| |

# End of study

|  |  |
| --- | --- |
| - 1. Date of cessation of participation (or death) | \|__\|__\| / \|__\|__\| / \|__\|__\|__\|__\| |
| - 1. Reason for participation ending | End of the protocol and all assessments carried out  Death, specify... ......................................  Lost to sight  Withdrawal of consent  Another reason, to specify:... .............................. .. |
